# Improved nearest-neighbor parameters for the stability of RNA/DNA hybrids under a physiological condition

**DOI:** 10.1093/nar/gkaa572

**Published:** 2020-07-14

**Authors:** Dipanwita Banerjee, Hisae Tateishi-Karimata, Tatsuya Ohyama, Saptarshi Ghosh, Tamaki Endoh, Shuntaro Takahashi, Naoki Sugimoto

**Affiliations:** FIBER (Frontier Institute for Biomolecular Engineering Research), Konan University, 7-1-20 Minatojima-Minamimachi, Chuo-ku, Kobe 650-0047, Japan; FIBER (Frontier Institute for Biomolecular Engineering Research), Konan University, 7-1-20 Minatojima-Minamimachi, Chuo-ku, Kobe 650-0047, Japan; FIBER (Frontier Institute for Biomolecular Engineering Research), Konan University, 7-1-20 Minatojima-Minamimachi, Chuo-ku, Kobe 650-0047, Japan; FIBER (Frontier Institute for Biomolecular Engineering Research), Konan University, 7-1-20 Minatojima-Minamimachi, Chuo-ku, Kobe 650-0047, Japan; FIBER (Frontier Institute for Biomolecular Engineering Research), Konan University, 7-1-20 Minatojima-Minamimachi, Chuo-ku, Kobe 650-0047, Japan; FIBER (Frontier Institute for Biomolecular Engineering Research), Konan University, 7-1-20 Minatojima-Minamimachi, Chuo-ku, Kobe 650-0047, Japan; FIBER (Frontier Institute for Biomolecular Engineering Research), Konan University, 7-1-20 Minatojima-Minamimachi, Chuo-ku, Kobe 650-0047, Japan; FIRST (Graduate School of Frontiers of Innovative Research in Science and Technology), Konan University, 7-1-20 Minatojima-Minamimachi, Chuo-ku, Kobe 650-0047, Japan

## Abstract

The stability of Watson–Crick paired RNA/DNA hybrids is important for designing optimal oligonucleotides for ASO (Antisense Oligonucleotide) and CRISPR (Clustered Regularly Interspaced Short Palindromic Repeats)–Cas9 techniques. Previous nearest-neighbour (NN) parameters for predicting hybrid stability in a 1 M NaCl solution, however, may not be applicable for predicting stability at salt concentrations closer to physiological condition (e.g. ∼100 mM Na^+^ or K^+^ in the presence or absence of Mg^2+^). Herein, we report measured thermodynamic parameters of 38 RNA/DNA hybrids at 100 mM NaCl and derive new NN parameters to predict duplex stability. Predicted Δ*G*°_37_ and *T*_m_ values based on the established NN parameters agreed well with the measured values with 2.9% and 1.1°C deviations, respectively. The new results can also be used to make precise predictions for duplexes formed in 100 mM KCl or 100 mM NaCl in the presence of 1 mM Mg^2+^, which can mimic an intracellular and extracellular salt condition, respectively. Comparisons of the predicted thermodynamic parameters with published data using ASO and CRISPR–Cas9 may allow designing shorter oligonucleotides for these techniques that will diminish the probability of non-specific binding and also improve the efficiency of target gene regulation.

## INTRODUCTION

The hybridization of RNA with complementary DNA occurs in many key steps of various significant biological processes, including in Okazaki fragmentation during DNA replication (1), in the transcription bubble (2), and during the reverse transcription process in the life cycle of human immunodeficiency virus (HIV) (3). RNA/DNA hybrids are also important for their therapeutic applications using ASO (Antisense Oligonucleotide) and CRISPR (Clustered Regularly Interspaced Short Palindromic Repeats)–Cas9 techniques. ASO (4) is based on RNase H mediated degradation of RNA in hybrid duplexes (5) and has been proved very beneficial in the treatment of gene expression instigating diseases (6,7). Recently, a genome editing CRISPR-Cas9 technology has been developed that is expected to be a useful tool for the treatment of genetic disorders (8–10). The efficiencies of both RNase-H and Cas9 endonucleases are largely affected by the sequences and stabilities of hybrid duplexes composed of the target gene with applied complementary oligonucleotides (11,12). Moreover, telomerase activity, which is related to the existence of prolonged genes in cancer cells, can be regulated by the stability of hybrid duplexes (13). Therefore, to elucidate the mechanism of different biological processes, as well as improve therapeutics, the precise prediction of the stability of RNA/DNA hybrids under physiological conditions is required.

An approach to predict the stability of nucleic acid secondary structures from their sequences was established using the nearest-neighbor (NN) model by Tinoco *et al.* (14,15). According to this model, the thermodynamic stability of the duplex with Watson–Crick base pairing can be determined by the summation of the free energy changes due to all adjacent NN base pair formations in the sequence and helix initiation. Following this model, our group previously introduced NN parameters for RNA/DNA hybrid duplexes (16) in a solution containing 1 M NaCl. Prediction of duplex stabilities was extensively studied in a standard 1 M NaCl solution (16–19); however, duplex stability in a lower salt concentration (∼100 mM) was also reported to be closer to that observed at physiological conditions (20–23). To derive the stability of nucleic acid duplexes (RNA, DNA and hybrid duplexes) in 100 mM NaCl solution from the estimated stability in 1 M NaCl solution, a simple method was reported by our group (21). The linear relationship between duplex stabilities in 1 M and 100 mM NaCl buffer solution (21) was proved useful for the rough estimation of duplex stability independent of sequence, length, or structure. However, several reports suggested that the effect of salt concentration on duplex stabilities depends on the sequence components (24,25). Equations for calculating the stabilities of DNA (22,26) and RNA duplexes (23) in 100 mM salt concentration have already been developed in which correction terms for sequences were incorporated. However, these equations are not applicable for RNA/DNA hybrids as the interactions of RNA/DNA hybrids with cations are different from that of RNA and DNA duplexes due to the different flexibilities and structures of hybrid duplexes (27). Recently, the prediction of the stability of only hybrid sequences in 100 mM NaCl solution was reported (28), where parameters were derived using theoretical optimization of melting temperatures (*T*_m_). This method is very useful and adequate for calculating the *T*_m_ of hybrid duplexes, however the method is not satisfactory for estimating other thermodynamic parameters (Δ*H*°, Δ*S*° and Δ*G*°_37_). Therefore, an improved prediction method is needed for hybrid duplexes under the physiological salt concentration.

In this study, we have measured the thermodynamics of 38 hybrid sequences in a buffer containing 100 mM NaCl and verified the accuracy of previous methods for predicting the stability of all sequences. We observed that several sequences specifically rG−dC- or rC−dG-rich hybrid sequences containing purine-rich RNA, as well as rA−dT- or rU−dA- rich hybrid sequences containing pyrimidine-rich RNA, could not be predicted well using the previous NN parameters (21). We found marked average differences between the measured and predicted values for Δ*G*°_37_ and *T*_m_ (10.7% and 4.9°C, respectively). To improve the prediction, we developed new NN parameters that significantly reduced the average differences for the measured and predicted Δ*G*°_37_ and *T*_m_ (2.9% and 1.1°C, respectively). Improved parameters can be used to predict not only the melting temperatures, but also the estimation of all thermodynamic parameters remarkably well for all hybrid sequences in 100 mM NaCl solution. For computing duplex stability of any sequence in 100 mM or in 1 M NaCl solution with ease, an open access website was created. We showed that hybrid stability under typical physiological salt conditions with various monovalent and divalent cations such as Na^+^, K^+^, Mg^2+^, Ca^2+^ (29) can be precisely predicted using the new parameters. Moreover, the individual effects of the monovalent and divalent cations on the stability of hybrid duplexes were discussed. We also demonstrated that the prediction of hybrid stabilities using our new parameters could be very useful for further developing of ASO and CRISPR-Cas9 techniques by enhancing the specificity and efficiency of the drugs.

## MATERIALS AND METHODS

### Materials and reagents

The RNA oligomers listed in Table 1 and their complementary DNA oligonucleotides were purchased from Japan Bio Service Co. at High Performance Liquid Chromatography (HPLC) grade. RNA and the complementary DNA sequences of different lengths and base compositions were used as model sequences in several experiments. TOF-MS (Time Of Flight Mass Spectrometer) and HPLC (High Performance Liquid Chromatography) spectra of the four RNA and DNA oligomers of the model hybrid sequences are included in Supporting Information (Supplementary Figure S1) to exhibit the purity of purchased oligonucleotides. Concentration of each oligonucleotide was determined from average absorbance in a higher temperature range (80–90°C) at 260 nm using the single-strand extinction coefficient (30). The RNA oligomers of Table 1 and their complementary DNA strands were mixed at an equimolar concentration to prepare corresponding RNA/DNA hybrids for this study and the RNA/DNA hybrid sequences are written here as rGCCGUGAG/dCTCACGGC for 5′rGCCGUGAG3′/5′dCTCACGGC3′. The chemicals like sodium chloride (NaCl), potassium chloride (KCl), magnesium chloride (MgCl_2_), calcium chloride (CaCl_2_), disodium hydrogen phosphate (Na_2_HPO_4_) and dipotassium hydrogen phosphate (K_2_HPO_4_) were purchased from Wako Pure Chemical Industries (Japan). Disodium ethylenediaminetetraacetate (Na_2_EDTA) and dipotassium ethylenediaminetetraacetate (K_2_EDTA) were purchased from Dojindo Molecular Technologies (Japan). All the chemicals were used as received.

**Table 1.** Thermodynamic parameters of RNA/DNA hybrid duplexes in 100 mM NaCl buffer solution

No.	RNA sequences^a^	Measured parameters in 100 mM NaCl solution^b^				Old prediction^d^		New prediction^e^	
		Δ*H*° kcal mol^−1^	*T*Δ*S*° kcal mol^−1^	Δ*G*°_37_ kcal mol^−1^	*T*_m_^c^ °C	Δ*G*°_37_ kcal mol^−1^	*T*_m_^c^ °C	Δ*G*°_37_ kcal mol^−1^	*T*_m_^c^ °C
1	GGUCGC	−42.6 ± 1.5	−36.0 ± 1.3	−6.6 ± 0.3	27.2	−6.0	20.8	−6.5	27.4
2	CGGACC	−55.0 ± 2.3	−48.6 ± 2.1	−6.4 ± 0.4	26.1	−6.1	21.4	−6.4	27.1
3a	GCCGUGAG	−72.5 ± 2.4	−63.4 ± 2.1	−9.1 ± 0.4	41.2	−7.5	32.2	−8.9	41.0
3b	GAGCCGUG	−78.1 ± 2.5	−69.1 ± 2.3	−9.0 ± 0.4	41.5	−7.5	32.2	−8.9	41.0
4a	GUCAGACU	−57.1 ± 1.4	−50.4 ± 1.2	−6.7 ± 0.2	29.7	−5.8	19.2	−6.7	30.1
4b	GACAGUCU	−54.2 ± 2.9	−47.3 ± 2.5	−6.9 ± 0.5	30.1	−5.8	19.2	−6.7	30.1
5	GAACUGCC	−66.7 ± 1.8	−59.4 ± 1.7	−7.3 ± 0.3	33.5	−7.1	30.1	−7.4	33.7
6	GGCAGUUC	−78.2 ± 3.7	−71.0 ± 3.4	−7.2 ± 0.5	33.8	−6.7	27.0	−7.4	34.0
7	GCGAUCGGA	−77.6 ± 2.8	−67.9 ± 2.4	−9.7 ± 0.5	43.5	−8.5	37.3	−9.9	45.2
8	GCCAGUAGG	−74.8 ± 2.3	−65.4 ± 2.0	−9.4 ± 0.4	42.6	−8.5	38.2	−9.7	43.7
9	GUUCAAUACG	−62.9 ± 2.3	−57.0 ± 2.1	−5.9 ± 0.3	27.5	−6.0	23.4	−6.3	29.6
10	AGGAUGACCG	−79.1 ± 1.5	−68.7 ± 1.3	−10.4 ± 0.3	45.9	−9.6	43.4	−10.9	48.0
11	CGCUUGUUAC	−76.9 ± 2.4	−69.9 ± 2.2	−7.0 ± 0.3	33.1	−6.7	27.1	−6.4	30.3
12	GUAACAAGCG	−81.6 ± 2.2	−72.9 ± 1.9	−8.7 ± 0.3	39.2	−7.8	33.5	−8.7	39.5
13	CACUUGUUAC	−73.9 ± 1.6	−68.0 ± 1.5	−5.9 ± 0.2	28.1	−5.8	22.1	−5.7	27.6
14a	AAUCUGGCCA	−57.8 ± 2.7	−48.7 ± 2.3	−9.1 ± 0.5	42.8	−8.8	39.2	−9.1	41.2
14b	AUGGCUCCAA	−64.5 ± 2.6	−55.6 ± 2.2	−8.9 ± 0. 4	40.1	−8.8	39.2	−9.1	41.2
15	GGGGAACAAGG	−110.5 ± 2.4	−96.6 ± 2.1	−13.9 ± 0.4	54.3	−12.1	54.3	−14.1	55.6
16	UUCACCUGGUC	−85.1 ± 1.9	−74.8 ± 1.7	−10.3 ± 0.3	45.3	−9.0	38.8	−10.4	45.5
17a	GGCAGGAAUCCG	−100.8 ± 3.0	−86.6 ± 2.6	−14.2 ± 0.5	56.8	−12.1	51.2	−14.2	56.5
17b	GGAAUCAGGCCG	−107.5 ± 4.3	−93.1 ± 3.8	−14.4 ± 0.7	56.3	−12.1	51.2	−14.2	56.5
18a	UAUCUUCCGAAU	−60.7 ± 1.7	−54.0 ± 1.5	−6.7 ± 0.3	30.2	−7.7	31.4	−7.0	32.9
18b	UAUCCUUCGAAU	−57.5 ± 1.2	−50.9 ± 1.1	−6.6 ± 0.2	29.6	−7.7	31.4	−7.0	32.9
19a	AAUGGAUUACAA	−83.1 ± 2.2	−75.3 ± 2.0	−7.8 ± 0.3	36.3	−8.2	33.9	−8.0	36.8
19b	AUUGGAUACAAA	−79.3 ± 3.6	−71.3 ± 3.2	−8.0 ± 0.5	36.2	−8.2	33.9	−8.0	36.8
20	CCUGGAAUCCAA	−85.1 ± 2.4	−74.0 ± 2.1	−11.1 ± 0.4	48.2	−9.9	42.7	−11.6	49.2
21	GGCUCAAUUGAC	−100.4 ± 2.0	−89.7 ± 1.8	−10.7 ± 0.3	45.2	−9.8	42.2	−10.8	46.2
22a	CGGCCUUGAUCC	−104.8 ± 4.0	−92.1 ± 3.5	−12.7 ± 0.6	51.9	−11.0	45.9	−12.2	51.1
22a	CGGAUUCCUGCC	−92.4 ± 2.6	−80.5 ± 2.3	−11.9 ± 0.4	50.3	−11.0	45.9	−12.2	51.1
23	UCCGAAUUAUCU	−81.5 ± 2.8	−73.6 ± 2.6	−7.9 ± 0.4	35.8	−7.7	31.4	−7.0	32.9
24	AGAUAAUUCGGA	−83.5 ± 2.4	−75.8 ± 2.2	−7.7 ± 0.3	35.5	−8.6	35.5	−8.6	38.9
25	GCUUCUCUCUUC	−73.1 ± 1.1	−66.4 ± 1.0	−6.7 ± 0.1	31.5	−7.7	31.9	−6.8	31.9
26	GAAGAGAGAAGC	−84.8 ± 4.1	−72.3 ± 3.5	−12.5 ± 0.7	54.0	−10.5	49.0	−12.6	52.0
27	UCGUUCUUGUCU	−77.8 ± 2.1	−70.0 ± 1.9	−7.8 ± 0.3	36.4	−7.4	30.1	−7.8	36.0
28	AGACAAGAACGA	−95.9 ± 2.1	−84.7 ± 1.8	−11.2 ± 0.3	47.6	−10.0	44.6	−11.5	48.1
29a	GUUAGCGUUACGC	−89.2 ± 3.1	−78.8 ± 2.7	−10.4 ± 0.5	45.0	−10.1	40.4	−11.0	45.9
29b	GCGUUUACGUAGC	−102.6 ± 1.1	−91.1 ± 1.0	−11.5 ± 0.2	47.8	−10.1	40.4	−11.0	45.9
30	UCACGUAGUCGUAU	−113.6 ± 4.4	−101.0 ± 3.9	−12.6 ± 0.7	49.8	−10.2	40.2	−12.6	50.7

^a^The designed hybrid duplex consists of a denoted RNA strand and its complementary DNA strand; the denoted RNA strand is represented as GCCGUGAG for 5′rGCCGUGAG3′. The pair of hybrid duplexes with identical nearest-neighbors are given by the number Xa and Xb (X is 3, 4, 14, 17, 18, 19, 22 and 29).

^b^All experiments were carried out in solutions containing 100 mM NaCl, 10 mM Na_2_HPO_4_, and 1 mM Na_2_EDTA (pH 7.0).

^c^The melting temperatures were determined at a total oligomer strand concentration of 8 μM.

^d^The hybrid stability in 100 mM NaCl buffer was predicted using Equations (4) and (5) (21) at the respective stability in 1 M NaCl buffer, determined using NN parameters (16).

^e^The hybrid stability in 100 mM NaCl buffer was predicted using new nearest-neighbor parameters (Table 2). The average prediction error ΔΔ*G*°_37_ (measured Δ*G*°_37_ − predicted Δ*G*°_37_) which is represented as percentage of error with respect to predicted Δ*G*°_37_ and Δ*T*_m_ (measured *T*_m_ − predicted *T*_m_) were calculated for old prediction as 10.7% and 4.9°C, respectively and for new prediction as 2.9% and 1.1°C, respectively.

### Circular dichroism (CD) measurements

CD spectra were obtained using a JASCO J-820 spectropolarimeter equipped with a temperature controller. All CD spectra were collected from 350 to 200 nm wavelengths with a scan rate of 50 nm min^−1^ in 0.1 cm path-length cuvettes. The cuvette holding chamber was continuously flushed with a stream of dry N_2_ gas to avoid water condensation forming on the cuvette exterior. All the spectra were measured at 20 μM total strand concentration in the phosphate buffer solution containing either 1 M or 100 mM NaCl.

### UV measurements

UV absorbance was measured using a Shimadzu 1800 spectrophotometer equipped with a thermoprogrammer. The melting curves (absorbance versus temperature curves) of the hybrid duplexes at 10–12 different oligonucleotide concentrations (varying ∼100-fold) were obtained at 260 nm using annealing from 90°C to 0°C, followed by heating from 0°C to 90°C at a rate of 0.5°C min^−1^. Water condensation on the cuvette exterior at a low temperature was avoided by flushing with a constant stream of dry N_2_ gas.

### Determination of thermodynamics for hybrid duplex formations

The thermodynamic parameters (Δ*H*°, Δ*S*°) for the formation of RNA/DNA hybrids were determined from *T*_m_^−1^ versus ln(*C_t_*/4) plot as described in a previous studies (16,31) using the following Equation (1) .

(1) $$\begin{equation*}{{T_{\rm m}}^{ - 1}} = R{\rm{ ln }}\left( {{C_t}/4} \right)/\Delta H^\circ + \Delta S^\circ /\Delta H^\circ \end{equation*}$$

where *R* is gas constant and *C_t_* is total strand concentration of the oligonucleotides. The parameters (Δ*H*°, Δ*S*°) were assumed to be temperature independent and determined using the van’t Hoff analysis as the transition equilibrium involves only two states (single strand and duplex). The RNA oligomers of Table 1 and their complementary DNA oligomers were previously reported to form hybrids showing two-state transition (16,32). Moreover, model RNA/DNA hybrids were examined here to show the two-state transition using CD spectra and UV melting profiles. The CD spectra were obtained using temperature induced unfolding CD assays carried out from 10°C to 90°C with an interval of 10°C for hybrid duplexes, and the CD spectra for each duplex were found to be passing through an isodichroic point. The CD assays of the six model hybrid duplexes of various lengths and base compositions are shown in Supplementary Figure S2. The presence of an isodichroic point in the temperature induced CD assays confirmed the existence of only two conformational states (33,34) for each sequence used in the study. The two-state transition for any duplex can also be extrapolated from the identical denaturation and renaturation profiles of UV melting experiments. The lack of hysteresis between the denaturation and renaturation UV melting curves of the hybrid duplexes (Supplementary Figure S2) were another indication of two-state transition between the single strands and the duplex (35,36). Therefore, the difference in heat capacities (Δ*C*_p_) of the single strand and duplex state was assumed to be zero as per standard practice (16–18,20,37). The melting temperature (*T*_m_) range of each duplex was maintained between 20–55°C to minimize extrapolation to 37°C. Although finite value of Δ*C*_p_ was also reported during the folding of nucleic acids (38), especially for the oligonucleotides with dangling ends (39), the temperature-dependent changes in Δ*H*° and Δ*S*° can counterbalance each other (38). Therefore, Δ*G*°_37_ and *T*_m_ values are relatively insensitive to the Δ*C*_p_ compared to Δ*H*° and Δ*S*° (40). The Δ*G*°_37_ were derived from Δ*H*° and Δ*S*° using equation (2).

(2) $$\begin{equation*}\Delta G{^\circ _{37}} = \Delta H^\circ - T\Delta S^\circ \end{equation*}$$

where *T* represents a temperature of 310.15 K used to derive free energy change at 37°C (Δ*G*°_37_). The standard error values for Δ*H*° and Δ*S*° (σ_Δ_*_H_*_°_ and σ_Δ_*_S_*_°_, respectively) were estimated here from the linearity of the *T*_m_^−1^ versus ln(*C_t_*/4) plots and those for Δ*G*°_37_ (σ_Δ_*_G_*_°37_) were calculated using the following equation (21).

(3) $$\begin{eqnarray*} {({\sigma _{\Delta G^\circ 37}})^2} &=& {({\sigma _{\Delta H^\circ }})^2} + {310.15^2}{({\sigma _{\Delta S^\circ }})^2} \nonumber \\ &&-\, 2 \cdot 310.15{\rm{ }}\left( {{R_{\Delta H^\circ ,{\rm{ }}\Delta S^\circ }}} \right){\sigma _{\Delta H^\circ }}{\sigma _{\Delta S^\circ }} \end{eqnarray*}$$

where *R*_Δ_*_H_*_°, Δ_*_S_*_°_ is the correlation coefficient between Δ*H*° and Δ*S*°.

### Calculation of nearest-neighbor parameters

According to the NN model, the thermodynamic parameters (Δ*H*°, Δ*S*° and Δ*G*°_37_) for non-self-complementary duplex formation consists of two terms: (i) a parameter for the helix initiation with the first base pair formation in the duplex; (ii) a parameter for helix propagation that is the summation of parameters for all subsequent base pairing. All 16 NN base pairs are presented in abbreviated form. For example, 5′rAG3′/5′dCT3′ is represented as rAG/dCT. The NN parameters were determined (Table 2) using a linear least-squares computer program (Supporting Information) (41). To determine appropriate parameters, the selection of the hybrid sequences were done carefully so that all the potential 16 NN base pairs present in an almost equal number of frequencies (16). The *T*_m_ at 8 μM total strand concentration was predicted from the parameters obtained (Δ*H*° and Δ*S*°) using Equation (1).

**Table 2.** Nearest-neighbor parameters of hybrid duplex in 100 mM NaCl buffer solution^a^

Sequence	Δ*H*°_NN_ kcal mol^−1^	Δ*S*°_NN_ cal mol^−1^K^−1^	Δ*G*°_37NN_ kcal mol^−1^
rAA/dTT	−7.8	−22.9	−0.7
rAC/dGT	−10.1	−27.3	−1.5
rAG/dCT	−9.4	−26.2	−1.3
rAU/dAT	−5.8	−17.5	−0.4
rCA/dTG	−9.8	−27.4	−1.2
rCC/dGG	−9.5	−24.8	−1.7
rCG/dCG	−9.0	−24.3	−1.4
rCU/dAG	−6.1	−17.9	−0.4
rGA/dTC	−8.6	−22.7	−1.5
rGC/dGC	−10.6	−27.7	−2.0
rGG/dCC	−13.3	−35.7	−2.3
rGU/dAC	−9.3	−25.5	−1.4
rUA/dTA	−6.6	−19.7	−0.5
rUC/dGA	−6.5	−16.3	−1.4
rUG/dCA	−8.9	−23.3	−1.6
rUU/dAA	−7.4	−24.3	0.2
init. rG−dC/rC−dG^b^	0	−4.9	2.0
init. rA−dT/rU−dA^c^	0	−7.0	2.6

^a^16 nearest-neighbour parameters and initiation for rG−dC or rC−dG pairing (init. rG−dC/ rC−dG) and initiation for rA−dT or rU−dA pairing (init. rA−dT/rU−dA) derived for RNA/DNA hybrid in 100 mM NaCl solution using the data of Table 1. The average errors estimated for Δ*H*°_NN_, Δ*S*°_NN_, and Δ*G*°_37NN_ in 100 mM NaCl solution were ±0.08 kcal mol^−1^, ±0.35 cal mol^−1^ K^−1^ and ±0.06 kcal mol^−1^ respectively.

^b^Initiation parameters for the duplexes that contains at least one rG−dC or rC−dG base pair in any terminal.

^c^Initiation parameters for the duplexes that contains only rA−dT or rU−dA base pairs in both terminal.

A website was developed to calculate the stability of any duplex by applying its sequence and salt concentration. In this website, we used reported parameters for DNA duplex (20), RNA duplex (18), and RNA/DNA hybrid in 1 M NaCl solution (16) for evaluating respective stability in 1 M salt concentration and the new NN parameters derived here (Table 2) for determining the stability of hybrid duplex in a physiological condition. To expand the scope of using additional parameters, we designed an option named ‘Manual’ in the website. The details about the website is given in the Supporting Information.

## RESULTS AND DISCUSSION

### Measurement of thermodynamic parameters for RNA/DNA hybrids

A total of 38 RNA sequences with different base compositions and oligomer lengths (6−14 mers) and their complementary DNA sequences were designed (Table 1) here to study thermodynamics of RNA/DNA hybrids. Parameters of Δ*H*°, *T*Δ*S*°, Δ*G*°_37_ and *T*_m_ of all the RNA/DNA hybrids were measured in a buffer solution containing 100 mM NaCl, 10 mM Na_2_HPO_4_, and 1 mM Na_2_EDTA (pH 7.0). The measured stabilities (Δ*G*°_37_ and *T*_m_) were compared with the corresponding stabilities, which were predicted using linear Equations (4) and (5), as proposed by Nakano *et al.* (21) (Table 1).

(4) $$\begin{equation*}\Delta G{^\circ _{37}}{\rm{ }}\left( {100\,{\rm{ mM}}} \right) = 0.63\,{\rm{ }}\Delta G{^\circ _{37}}\left( {1{\rm{ M}}} \right) - 1.667\end{equation*}$$

(5) $$\begin{equation*}{T_{\rm{m}}}{\rm{ }}\left( {100\,{\rm{ mM}}} \right) = 0.876\,{\rm{ }}{T_{\rm{m}}}{\rm{ }}\left( {1{\rm{ M}}} \right) - 5.148\end{equation*}$$

**Figure 1.**
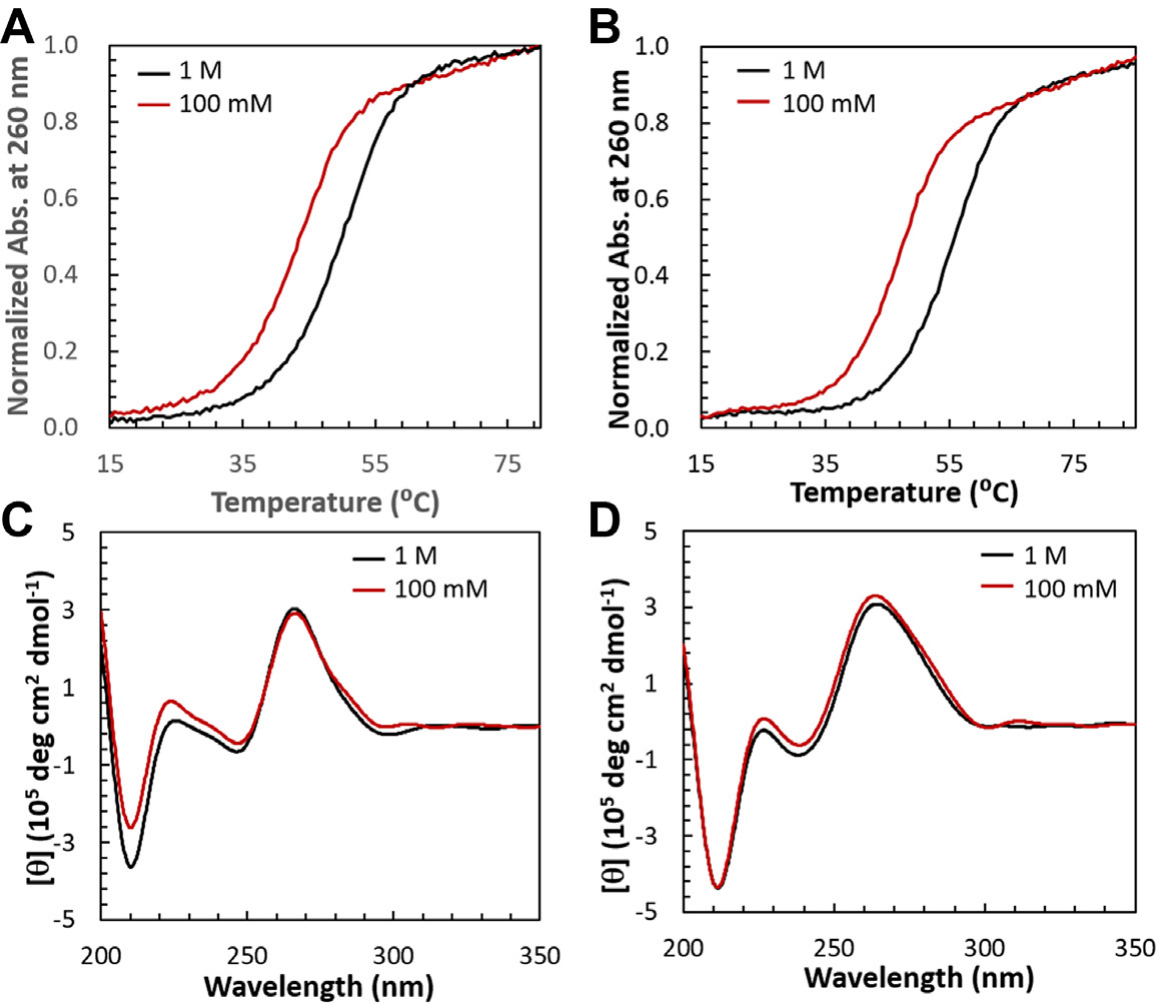
Normalised UV melting curves of hybrid duplexes for 8 (rGCCAGUAGG/dCCTACTGGC) (**A**) and 10 (rAGGAUGACCG/dCGGTCATCCT) (**B**). Circular dichroism spectra of hybrid duplexes for 8 (**C**) and 10 (**D**). All UV melting curves and CD spectra were measured at total strand concentration of 8 and 20 μM, respectively, in two buffer solutions containing 1 M (black) and 100 mM (red) NaCl.

The prediction errors, which represented the differences between the measured and predicted values (Table 1) of Δ*G*°_37_ [ΔΔ*G*°_37_ = measured Δ*G*°_37_ − predicted Δ*G*°_37_] and *T*_m_ [Δ*T*_m_ = measured *T*_m_ − predicted *T*_m_] were estimated. The average ΔΔ*G*°_37_ and Δ*T*_m_ values for all the hybrid duplexes (10.7% and 4.9°C, respectively) were significantly higher than the average prediction error reported (5.7% and 2.4°C, respectively) (21). Fifteen sequences (**1**, **3a**, **3b**, **4a**, **4b**, **7**, **12**, **16**, **17a**, **17b**, **20**, **22a**, **26, 29b** and **30**) among the 38 sequences (Table 1) showed large prediction errors for both Δ*G*°_37_ and *T*_m_ (≥10% and ≥5°C, respectively), along with several sequences that showed large prediction errors for either Δ*G*°_37_ (**8**, **15**, **18a**, **18b, 24** and **25**) or *T*_m_ (**6**, **11**, **13** and **27**). The large values of prediction errors for several hybrid sequences will need reconsideration for the prediction method. The duplex stabilities at a salt concentration of 100 mM were estimated using Equations (4) and (5) (21) based on the stabilities calculated in 1 M NaCl using the previous NN parameters (16). Therefore, the discrepancies between the measured and the predicted stabilities may lie either in the first step of prediction using the NN parameters in 1 M NaCl or in the second step of calculation using the linear Equations (4 and 5). For the first step, we compared the experimentally observed and predicted stabilities (Δ*G*°_37_ and *T*_m_) in 1 M NaCl solutions (Supplementary Table S1) of the several model hybrid duplexes of different lengths and base compositions selected from Table 1. As a result, the experimental and predicted stabilities (Δ*G*°_37_ and *T*_m_) of the selected sequences displayed a high similarity (Supplementary Table S1) with low values of average prediction errors (4.6% and 1.7°C, respectively), indicating a high accuracy of the stability prediction using the previous NN parameters (16) in 1 M NaCl. Reductions in duplex stabilities were prominent from the Δ*G*°_37_ and *T*_m_ values measured in 1 M (Supplementary Table S1) and 100 mM NaCl (Table 1) solutions. The thermodynamic parameters of several hybrid duplexes in 1 M and 100 mM salt concentration reported previously and also obtained by our experimental measurements indicated that both Δ*H°* and Δ*S°* were affected by salt concentration. However, the destabilization of hybrid duplexes resulting from the reduction of salt concentration from 1 M to 100 mM were induced by the greater entropic effect. This observation also supports previous reports about the effect of salt on duplex stability (42,43). Although the error of measurements for Δ*H°* and Δ*S°* were much higher with respect to that for Δ*G*°_37_ and *T*_m_. Thus we compared here the measured and predicted values of Δ*G*°_37_ and *T*_m_ only. The stability reductions from 1 M to 100 mM salt concentration are demonstrated by the typical melting curves of the two model hybrid duplexes rGCCAGUAGG/dCCTACTGGC (**8**) and rAGGAUGACCG/dCGGTCATCCT (**10**) in 1 M and 100 mM NaCl solutions (Figure 1A and B). However, the CD spectra of the two model hybrid sequences (**8** and **10**) and also other sequences (Table 1) obtained at 4°C in both salt concentrations indicated that there were no evident changes in the spectra between the 1 M and 100 mM NaCl solutions (Figure 1C, D, and Supplementary Figure S3). The results suggested that the deviations in the calculated stabilities in 100 mM NaCl were not due to any unexpected conformational changes of the hybrid duplexes caused by the reduction of NaCl concentration. The prediction in the 1 M salt concentration is highly accurate, but the accuracy is low in the 100 mM salt condition, so there is scope for improvement in the second step of the prediction.

To determine the limitations of Equations (4) and (5), we especially chose eight model RNA/DNA hybrids from Table 1 containing different base compositions. The *T*_m_s for the selected hybrid duplexes at 8 μM strand concentration were measured in solutions with varying NaCl concentrations (from 1 M to 50 mM) and plotted against log [Na^+^] (Supplementary Figure S4). The *T*_m_s of each hybrid duplex showed a linear dependency on log [Na^+^] at the range of 50 mM to 1 M. The slope and intercept values of each straight line varied from sequence to sequence. The large slope values indicate that the destabilization of hybrid due to reduction of Na^+^ concentration is significant. For example, the duplexes rGCCAGUAGG/dCCTACTGGC (**8**) and rAUGGCUCCAA/dTTGGAGCCAT (**14b**) had almost the same *T*_m_ in 1 M salt concentration (Supplementary Figure S4); however, they showed different slopes and hence different *T*_m_ at a salt concentration of 50 mM due to the presence of different base compositions. Similar results were observed for the duplexes rCGCUUGUUAC/dGTAACAAGCG (**11**) and rUAUCUUCCGAAU/dATTCGGAAGATA (**18a**) in Supplementary Figure S4. The hybrid duplex rGCCGUGAG/dCTCACGGC (**3a**) showed a lower slope, such that the order of the stabilities between **8** and **3a** were reversed at salt concentrations of 50 mM and 1 M (Supplementary Figure S4). These results clearly indicate that the decreasing order of stability for hybrid duplex at a salt concentration from 1 M to 50 mM is dependent on the sequence components (the rG−dC or rC−dG and rA−dT or rU−dA contents in hybrid duplex, as well as the purine and pyrimidine bases in the RNA and DNA strands). Notably, with an increase in the rG−dC or rC−dG content in the hybrid duplexes and the purine base in RNA, the slope values of the linear plots tended to be significantly small (Supplementary Figure S4). Since we only considered short oligonucleotides (8–12 mer), the effect of length was assumed to be insignificant. These results suggested that hybrid stability in 100 mM NaCl solution can not be estimated with significant accuracy using the linear equations due to the impact of base composition on the hybrid stability. Therefore, an improved prediction method is required for hybrid stability including the effect of base composition.

### Improvement of prediction for hybrid stability by linear equations

The effect of base components on the stability of hybrid duplexes in 100 mM NaCl solution were thoroughly studied here to improve prediction method of hybrid stability. As shown in Table 1, the measured stabilities for the rG−dC- and rC−dG-rich duplexes containing purine-rich RNA strands were much higher than the corresponding calculated stabilities. On the contrary, the measured stabilities of the rA−dT- or rU−dA-rich hybrids containing pyrimidine-rich RNA strands were lower than their predicted stabilities. Previous reports have suggested that the stabilities of hybrid duplexes in a low salt concentration is very sensitive toward purine and pyrimidine base asymmetries in the RNA or DNA strands (44,45). It was also suggested that rG−dC- or rC−dG-rich duplexes are much more stable than rA−dT- or rU−dA-rich duplexes at lower salt concentrations (44,45). To determine the individual effect of each sequence component on the hybrid stability more clearly, we defined two sequence factors, excess rG−dC or rC−dG content in duplex [ƒ(G−C)] and excess purine base in RNA [ƒ(rPu)]: ƒ(G−C) = (*N*_G−C_ − *N*_A−U/T_)/*N*_total_ and ƒ(rPu) = (*N*_pu_ − *N*_py_)/*N*_total_, where *N*_G−C_ is the amount of rG−dC or rC−dG content in the duplex, *N*_A−U/T_ is amount of rA−dT or rU−dA content in the duplex, *N*_pu_ is the number of purine bases in RNA strand, *N*_py_ is the number of pyrimidine bases in RNA strand, and *N*_total_ is the oligomer length. Based on the sequence compositions, we selected two sets of sequences (Set 1 and Set 2) using the sequences designed in this study and sequences from previous reports (Supplementary Table S2) (45,46). Set 1 included hybrid duplexes consisting of an equal number of rG−dC or rC−dG and rA−dT or rU−dA in the duplex [ƒ(G−C) = 0] but different ƒ(rPu) values. In contrast, Set 2 included hybrid duplexes consisting of equal numbers of purine and pyrimidine bases in RNA [ƒ(rPu) = 0] but different ƒ(G−C) values. The individual effect of ƒ(G−C) or ƒ(rPu) was evaluated by plotting the measured Δ*G*°_37_ (100 mM)/*N*_total_ of the sequences in Set 2 against ƒ(G−C) (Supplementary Figure S5A) or those of the sequences in Set 1 against ƒ(rPu) (Supplementary Figure S5B), respectively. For the plots, we used Δ*G*°_37_ (100 mM)/*N*_total_ instead of Δ*G*°_37_ (100 mM) to minimize the effect of oligomer length on the duplex stabilities. Both plots clearly indicated a decrease of Δ*G*°_37_ (100 mM)/*N*_total_ with increasing sequence factors ƒ(G−C) and ƒ(rPu), suggesting that both sequence factors affect the hybrid stability individually in 100 mM NaCl solution. Consequently, the hybrid sequences containing both factors as positive [ƒ(G−C) > 0 and ƒ(rPu) > 0] or as negative [ƒ(G−C) < 0 and ƒ(rPu) < 0] showed a large deviation of the measured stabilities from the predicted ones, due to the fact that the linear Equations (4) and (5) do not contain correction terms for the sequence factors. Therefore, an improvement of these linear Equations (4 and 5) is required in order to predict the hybrid stability in 100 mM NaCl with significant accuracy.

**Figure 2.**
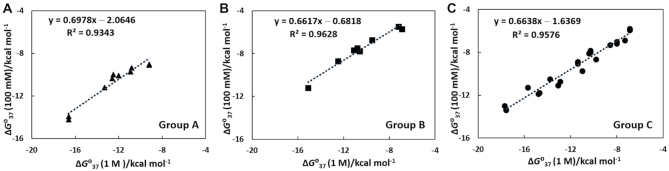
The plot of measured *ΔG*°_37_ in 100 mM NaCl versus predicted *ΔG*°_37_ in 1 M NaCl using the data of (**A**) hybrid duplexes under Group A (▴), (**B**) hybrids under Group B (▪) and (**C**) hybrid sequences under Group C (•) selecting the sequences from Table 1 and Supplementary Table S2.

Initially, we categorized the hybrid sequences into three groups depending on the prediction errors ΔΔ*G*°_37_ and Δ*T*_m_ (Table 1) and the sequence factors ƒ(G−C) and ƒ(rPu) (Supplementary Table S2). The hybrid sequences with measured stabilities that were higher than their predicted stabilities (Table 1) generally contained higher levels of rG−dC or rC−dG content in the duplex (ƒ(G−C) > 0) and higher levels of purine bases in the RNA (ƒ(rPu) > 0), such as the oligonucleotides rGGCAGGAAUCCG /dCGGATTCCTGCC (**17a**); we categorized these most stable hybrid sequences as Group A. In contrast, the hybrids that had measured stabilities that were lower than the predicted stabilities (Table 1) generally contained lower levels of rG−dC or rC−dG in the duplex (ƒ(G−C) < 0) as well as lower levels of purine bases in the RNA (ƒ(rPu) < 0), such as rUAUCUUCCGAAU/dATTCGGAAGATA (**18a**); these less stable hybrid sequences were allocated to Group B. Finally, Group C was comprised of all of the other mixed sequence contents, such as a higher rG−dC or rC−dG content in the duplex and fewer purine bases in the RNA, i.e. rCGGAUUCCUGCC/dGGCAGGAATCCG (**22a**), a lower rG−dC or rC−dG content in the duplex with more purine bases in the RNA, i.e. rAAUGGAUUACAA /dTTGTAATCCATT (**19a**), or equal distributions of rG−dC or rC−dG and rA−dT or rU−dA content in the duplex and the same purine and pyrimidine bases in the RNA (ƒ(G−C) = 0 and ƒ(rPu) = 0), i.e. rAUGGCUCCAA/dTTGGAGCCAT (**14b**). The stabilities of the hybrid duplexes under Group C (**14b**, **19a**, and **22a**) could be predicted more precisely using Equations (4) and (5) (Table 1) than for Group A (**17a**) or Group B (**18a**). The measured Δ*G*°_37_ in 100 mM NaCl versus predicted Δ*G*°_37_ in 1 M salt were plotted separately for the hybrid sequences of all three groups (Group A, Group B, and Group C). The obtained independent plots showed a good linear correlation (Figure 2). The linear equations corresponding to the hybrid sequences of the above-mentioned groups are as follows (Equations 6–8):

(6) $$\begin{eqnarray*} {\rm{Group\, A}}:{\rm{ }}\Delta G{^\circ _{37}}{\rm{ }}\left( {100\,{\rm{ mM}}} \right) &=& 0.698\,{\rm{ }}\Delta G{^\circ _{37}}{\rm{ }}\left( {1{\rm{ M}}} \right) \nonumber \\ && -\, 2.065 \end{eqnarray*}$$

(7) $$\begin{eqnarray*} {\rm{Group\, B}}:{\rm{ }}\Delta G{^\circ _{37}}{\rm{ }}\left( {100\,{\rm{ mM}}} \right) &=& 0.662\,{\rm{ }}\Delta G{^\circ _{37}}\left( {1{\rm{ M}}} \right) \nonumber \\ && -\, 0.682 \end{eqnarray*}$$

(8) $$\begin{eqnarray*} {\rm{Group\, C}}:{\rm{ }}\Delta G{^\circ _{37}}{\rm{ }}\left( {100\,{\rm{ mM}}} \right) &=& 0.664\,{\rm{ }}\Delta G{^\circ _{37}}{\rm{ }}\left( {1{\rm{ M}}} \right) \nonumber \\ && - \, 1.637 \end{eqnarray*}$$

When we plotted the measured Δ*G*°_37_ in 100 mM NaCl versus predicted Δ*G*°_37_ in 1 M salt using all the hybrid sequences (Supplementary Table S2) as suggested in previous method (21), a correlation was found (Supplementary Figure S6), although that was not satisfactory. A similar trend was also observed for the plot of measured *T*_m_ in 100 mM NaCl versus predicted *T*_m_ in 1 M salt (data not shown). The linear equation corresponding to Group C (Equation 8) was very similar to Equation (4), hence the stability of the sequences from Group C could be predicted well using the previous linear equation. However, the other two linear equations, corresponding to Group A and Group B (Equations 6 and 7, respectively), were different, mainly due to the large differences between the intercept values. Therefore, we assumed that the effect of salt concentration on the stability of hybrid duplexes belonging to Group C is less dependent on the sequence factors compared to that of Group A and Group B. Lesnik and Freier proposed a relationship between the helix conformation and thermodynamic stability of hybrid duplexes (45). Comparing the CD spectra (Supplementary Figure S3) with the reported structural analysis (45,46), it was observed that the hybrid duplexes of Group A generally show A-like conformation which is very similar to RNA duplexes. The conformations of hybrid duplexes belonging to Group B are significantly deviated from the A-like conformation. Hence, it is possible that the two different conformations were affected by the salt concentration in different manners due to the differences in their Na^+^ ion uptake during duplex formation. As hydration has a significant role in duplex formation, it is also possible that changes in water activity due to the change of salt concentration could affect duplex stability. However, the change of water activity by changing salt concentration from 1 M to 100 mM was reported to be small (22). Furthermore, the hybrid stability of the sequence which belongs to these groups could not be predicted by one linear equation, therefore at least three different linear equations for the three groups of hybrid duplexes would be needed. The three linear Equations (6)–(8) can be used to calculate the hybrid stabilities with a better accuracy than that with Equation (4), although no correction term for the sequence was included. The incorporation of correction factor could improve the prediction ability; however, the equation will no longer be linear and will become more complicated. Therefore, we required a simple and precise prediction method for hybrid stabilities under physiological conditions able to evaluate the effect of the sequence on stability. The NN model can explain the individual contribution of changes in enthalpy, entropy, or free energy for the formation of each subsequent base pair. Therefore, problems arising in the stability prediction due to the effect of sequence could be solved easily, such that the free energy change as well as the melting temperature for duplex formation could be evaluated more precisely.

### Improvement of prediction for hybrid stability using new nearest-neighbor parameters

The NN hypothesis assumes that duplexes with identical NN base pairs have identical melting behaviour and similar thermodynamic parameters for duplex formation. Therefore, to derive NN parameters for RNA/DNA hybrids in 100 mM NaCl solution, it is essential to verify the validity of the NN model under the same condition. For this purpose, two hybrid duplexes (**3a** and **3b**) with identical NNs were chosen from Table 1.

rGCCGUGAG/dCTCACGGC (**3a**) = rGC /dGC + rCC/dGG + rCG/dCG + rGU/dAC + rUG/dCA + rGA /dTC + rAG/dCT = rGAGCCGUG/dCACGGCTC (**3b**)

The melting curves and the plot of *T*_m_^−1^ versus ln (*C*_t_/4) were compared for the pair of hybrid duplexes **3a** and **3b** (Supplementary Figure S7), which showed similar melting behavior and a similar *T*_m_^−1^ versus ln(*C*_t_/4) plot. A total of eight pairs of hybrid duplexes with identical NNs were given as Xa and Xb (X = **3**, **4**, **14**, **17**, **18**, **19**, **22** and **29**) in Table 1. The average differences between the measured parameters (Δ*H*°, *T*Δ*S*°, Δ*G*°_37_ and *T*_m_) for all the pairs (Xa and Xb) were 8.3%, 9.2%, 3.5% and 1.1°C, respectively. Similar range of differences in the measured thermodynamic parameters (7.7%, 8.2%, 6.5% and 2.3°C, respectively) for the hybrid sequences with identical NNs were reported as a result of the experimental error (16) in the testing of the validity of NN model for hybrid duplexes in 1 M NaCl solution. Therefore, the hybrid duplexes follow the NN model even in 100 mM NaCl solution.

The NN parameters of Δ*H*°, Δ*S*°, and Δ*G*°_37_ (Δ*H*°_NN_, Δ*S*°_NN_ and Δ*G*°_37NN_, respectively) for the RNA/DNA hybrid in 100 mM NaCl buffer solution were obtained using the linear least square computer programming and the derived parameters for the 16 NN base pairs and two initiation for rG−dC or rC−dG and for rA−dT or rU−dA formations are provided in Table 2. In the improvement of NN parameters for hybrids, we have tried to incorporate an extra term for terminal rA−dT or rU−dA since terminal A−U base pair was introduced previously to improve the stability prediction of duplexes (19), and the term was applied to define the difference in the number of H-bonding between G−C and A−U base pairs at the terminals. However, the incorporation of the terminal rA−dT or rU−dA did not result any further improvement in the predictions here. The two different initiation terms corresponding to the rG−dC or rC−dG and the rA−dT or rU−dA formations are perhaps already compensating for the effect of extra H-bonding of terminals in hybrid stability. The 16 NN parameters derived in this study have a major enthalpy contribution in helix propagation and a major entropy contribution in helix initiation. For example, in the formation of the subsequent base pairs rAA/dTT in 100 mM NaCl buffer at 37°C, the enthalpy contribution (Δ*H*°_NN_) is −7.8 kcal mol^−1^, while the entropy contribution (*T*Δ*S*°_NN_) is −7.1 kcal mol^−1^, which is less than the enthalpy contribution. To derive the initiation parameters, we assumed that the helix initiations were negatively controlled by the entropy factor, due to the restriction of individual translational and rotational movements of the single strands by converting two particles into one. Hence, the changes in the enthalpy for both initiation terms were assumed to be zero (20), which also minimizes the error for all other Δ*H*°_NN_ parameters. The comparison between the NN parameters in 1 M (Supplementary Table S3) and 100 mM NaCl (Table 2) solution derived by the same algorithm clearly indicated an increment in Δ*G*°_37NN_ from 1 M to 100 mM NaCl solution and the increase of Δ*G*°_37NN_ be influenced by the sequence component of the respective NN. For example, the Δ*G*°_37NN_ values for the NNs containing higher levels of purine residues in the RNA [ƒ(rPu) > 0], such as rAA/dTT and rAG/dCT were increased by only 0.3 kcal mol^−1^. On the contrary, the increments in Δ*G*°_37NN_ for the NNs of rUU/dAA, and rCU/dAG containing complementary pyrimidine-rich RNA [ƒ(rPu) < 0] were large (0.6 kcal mol^−1^ and 0.7 kcal mol^−1^, respectively). Moreover, the NNs containing higher levels of rG−dC or rC−dG [ƒ(G−C) > 0] and purine-rich RNA [ƒ(rPu) > 0], such as rGG/dCC was found to have higher stability (Δ*G*°_37NN_) in 100 mM NaCl solution (−2.3 kcal mol^−1^), whereas the NN (rUU/dAA) with the lowest content of rG−dC or rC−dG [ƒ(G−C) < 0] and pyrimidine-rich RNA [ƒ(rPu) < 0] was the least stable. These observations confirmed the sequence-dependent reduction in stability as a result of a decrease in salt concentration, which correlated well with previous reports (47–50). To reduce the effort for calculating hybrid stability in 100 mM NaCl condition by our parameters (Table 2), we designed the website located at https://drive.google.com/open?id=1xF0TianMpQ6rwszN9gsweu7C5Wnzq0kF. We can also use the website to evaluate hybrid stability and the stability of DNA and RNA duplexes in 1 M NaCl solution using the reported parameters (16,18,20).

Our parameters were able to predict the stability of designed 38 RNA/DNA hybrids (Table 1) adequately, with average values of prediction error for Δ*H*°, Δ*S*° and Δ*G*°_37_ of 9.0%, 10.1% and 2.9%, respectively, and for *T*_m_ of only 1.1°C. The prediction of stability for the hybrid duplex was much improved by the obtained parameters compared to the linear Equations (4 and 5) (21). Even the new NN parameters can better predict all thermodynamic parameters of hybrid duplex than the recently published parameters by Barbosa *et al.* (28). The parameters obtained from the *T*_m_ optimization method (28) can be used to accurately estimate the *T*_m_ of our 38 designed sequences with the prediction error value only 2.2°C, however the prediction error for Δ*H*°, Δ*S*° and Δ*G*°_37_ came out much higher (20.3%, 22.2% and 6.5%, respectively) compared to the prediction error by our parameters. The reason behind the large prediction errors for Δ*H*°, Δ*S*° and Δ*G*°_37_ by the computational method may be due to the only optimization of *T*_m_ values reported in previous studies (21,45,46), while no optimization was done for other thermodynamic parameters. Although melting temperature is sensitive to the strand concentration, thermodynamic parameters (Δ*H*°, Δ*S*° and Δ*G*°_37_) are not. We could predict the *T*_m_ values at any strand concentration for a duplex using these parameters. Therefore, thermodynamic parameters have significant importance and optimization of all the parameters is required. Moreover, large differences were observed between our parameters (Table 2) and the parameters derived by Barbosa *et al.* For example, our parameters showed the increasing order of Δ*G*°_37NN_ for NNs as rGC/dGC < rCC/dGG < rCU/dAG, which was expected and observed for the parameters in 1 M NaCl (16). However, according to the parameters by Barbosa *et al.*, the Δ*G*°_37NN_ values of these three NNs were the same. Besides the formation of the subsequent base pairs rGA/dCT with purine-rich RNA has been reported by Barbosa *et al.* as less stable than that of rCA/dGT which is also not relevant based on our experimental observations. In that theoretical study (28), Barbosa *et al.* derived parameters via optimization of *T*_m_ of reported sequences (21,45,46) containing an unequal number of frequencies for 16 NNs. By contrast, in our study, to derive new parameters, we selected sequences such a way that the number of all NN base pairs were as equal as possible. Therefore, the new parameters can accurately predict all the thermodynamic parameters of hybrid duplexes under a physiological condition.

Since the NN parameters were derived here using the measured thermodynamic parameters of the 38 sequences (Table 1), the new NN parameters would clearly be able to predict the stabilities of the 38 hybrid sequences with more accuracy. Therefore, we compared the stabilities of a new set of 26 hybrid duplexes previously measured by other groups (21,45,46) with the stabilities predicted using the new parameters (Supplementary Table S4). Comparison showed that the new parameters can predict well the hybrid stabilities, as the average values of prediction error (ΔΔ*G*°_37_ and Δ*T*_m_) for the 26 sequences came out at 8.5% and 2.7°C, respectively. Previously, NN parameters were also improved which estimate duplex stabilities with similar prediction errors (20,37). The new NN parameters can only predict hybrid stability of oligonucleotides like the previous NN parameters that were reported for stability prediction of different nucleic acid duplexes (16,20). We have tested the parameters by predicting the stabilities of a total of 64 hybrid sequences with lengths between 6 and 16 mer and obtained reasonable average prediction errors for Δ*G*°_37_ and *T*_m_ (4.7%, and 1.6°C, respectively). Oligomers shorter than 6 mer generally give a *T*_m_ much lower and too far from 37°C which violates the assumption for determining Δ*H°* and Δ*S°*, and since the determination of *T*_m_ depends on the upper and lower baselines of the melting curves, obtaining a reliable *T*_m_ from the melting curves was in some cases difficult for much shorter and longer sequences. On the other hand, longer oligomers have a higher probability to form intramolecular folding that induces a non-two-state transition from single strand to duplex (51).

### Prediction of hybrid stability in physiological salt condition

In the living cell, apart from Na^+^ ions, monovalent cations like K^+^ and various divalent cations are also present. The concentrations of Na^+^ and K^+^ ions are reversed for the intracellular and extracellular condition. Potassium ion concentration is dominating inside a resting mammalian cell, whereas extracellular fluid mainly contains Na^+^ ions (29). As both mentioned monovalent cations are dominating in respective environments and they play important functions and regulations, it is also important to know the effect of KCl along with the NaCl salt concentration on the stability of hybrid duplexes. Therefore, we also studied hybrid stabilities in 100 mM KCl. Sequences from Group A (**10**, **17a**), Group B (**18a**) and Group C (**9**) were selected from Table 1 to verify the efficacy of the new parameters in predicting hybrid stabilities even in 100 mM KCl solution. We determined the thermodynamic parameters of the hybrid sequences (**9**, **10**, **17a** and **18a**) in a buffer solution containing 100 mM KCl, 10 mM K_2_HPO_4_, and 1 mM K_2_EDTA (pH 7.0), and compared them with the stabilities predicted using the new parameters (Table 2) in Supplementary Table S5. The stabilities (Δ*G*°_37_ and *T*_m_) measured in the buffer containing 100 mM KCl were plotted (Supplementary Figure S8) against that measured in the buffer containing 100 mM NaCl (Table 1). All four points in the plots of Δ*G*°_37_ and *T*_m_ in Supplementary Figure S8 appeared on the diagonal lines suggesting a similar effect of monovalent Na^+^ and K^+^ cations on hybrid stabilities. This observation indicates that the duplexes were stabilized to the same extent in the presence of both cations (Na^+^ and K^+^) in the same concentration as reported earlier (21,26,52). A comparison (Supplementary Table S5) of the measured stabilities of the hybrid duplexes in 100 mM KCl and the corresponding stabilities predicted using the new parameters clearly displayed that the new parameters (Table 2) can predict the stability (Δ*G*°_37_ and *T*_m_) of hybrid duplexes in physiological conditions even in the presence of K^+^ ions with average prediction errors of 7.3% and 2.4°C, respectively.

Living cell also contains various divalent cations. To evaluate hybrid stability in realistic physiological salt condition, we further measured the stabilities of hybrids in a solution containing a composition of different cations similar to mammalian intracellular environment (29). A solution containing 120 mM KCl, 10 mM NaCl, 1 mM MgCl_2_, 0.2 μM CaCl_2_ and 10 mM K_2_HPO_4_ (pH 7.0) was selected to mimic the intracellular conditions of a typical mammalian cell. We selected three sequences from three different groups (Group A, Group B, and Group C) to measure hybrid stabilities under the above mentioned physiological condition.

To examine the improvement of accuracy of the stability prediction by our parameters, we compared hybrid stabilities measured in a typical physiological condition with that obtained for the corresponding sequences in 1 M and 100 mM NaCl solutions using melting curves (Supplementary Figure S9). The comparison indicated that hybrid stability measured in a typical physiological condition is close to that obtained in 100 mM NaCl, however far from that obtained in 1 M NaCl solution (Supplementary Figure S9), and thus can be better predicted from the improved parameters determined in 100 mM NaCl solution. Excluding monovalent cations like Na^+^ and K^+^, Mg^2+^ is the most abundant cation present in cells with a varying concentration depending on cell type and the specific compartment of the cell. The concentration of free Mg^2+^ is maintained between 0.4 to 1.2 mM (53). The effect of Mg^2+^ ion on the stability of DNA duplex is dependent on the concentration of not only Mg^2+^ ion but also Na^+^ ion in the solution (21,26). In a solution containing 100 mM NaCl, if the concentration of Mg^2+^ ion increases above 1 mM, it can stabilize duplexes as only Mg^2+^ can stabilize in the absence of NaCl. On the contrary, if Mg^2+^ concentration remains below 1 mM in a solution of 100 mM NaCl, it can stabilize duplexes as only Na^+^ can stabilize in absence of Mg^2+^ ions (21,26). Therefore, a mixture of 1 mM MgCl_2_ and 100 mM NaCl in 10 mM Na_2_HPO_4_ buffer solution (pH 7.0) was chosen here to study the effect of Mg^2+^ ions in the presence of Na^+^ ions on the stability of different groups of hybrid sequences. We measured the thermodynamic parameters of the three selected hybrid sequences (**17a**, **23**, and **21** from Table 1) from the three different groups (Group A, Group C and Group B, respectively) and compared their stabilities in 100 mM NaCl buffer solution with and without 1 mM Mg^2+^ ion concentration (Supplementary Table S6). The stabilities (Δ*G*°_37_ and *T*_m_) measured in both buffer solutions were plotted against each other (Supplementary Figure S10). The stabilities of all hybrid duplexes were close to the diagonal line of the plots, revealing that the new parameters can be applied to predict hybrid stability in a solution of 100 mM NaCl containing up to 1 mM of Mg^2+^ ions. However, the stabilities of the sequence (**17a** in Supplementary Table S6) from Group A was observed to deviate slightly (Supplementary Figure S10A). In the presence of Mg^2+^ ions, the sequence from Group A is slightly more stabilized compared to the other sequences from Group C and Group B. Interestingly, the hybrid duplexes of Group A contain rG–dC or rC–dG rich hybrid sequences with purine rich RNA. The stronger stabilization of the hybrid sequences of Group A by Mg^2+^ ions can be explained by a previous report which suggests that Mg^2+^ preferentially binds with RNA duplexes (54), as well as with the N7 of purine bases (55). We already observed that an RNA/DNA hybrid having purine-rich RNA and belonging to Group A obtains a more A-like conformation, similar to an RNA duplex (46,56), compared to sequences belonging to Group B and Group C. The preferential binding of Mg^2+^ to the deeper major groove of the A-form or A-like conformation over the wider major groove of the B-form or B-like conformation was already known (54). Thus the differences in stabilities for different groups of hybrid sequences in the presence of Mg^2+^ can be attributed to the different helical structures of hybrid duplexes. However, in the presence of 1 mM Mg^2+^ ion the differences were not considerable. Since the hybrid duplexes from the three groups exhibited similar stabilities in the absence and presence of 1 mM Mg^2+^ ion in 100 mM NaCl solution, our parameters can be applied to predict the stability of hybrid duplexes in physiological salt conditions even in the presence of divalent cations.

### Prediction of hybrid stability in therapeutic application

Improvements in stability prediction of hybrid sequences could be used in therapeutic applications. Hybrid sequences and their stabilities are important factors for inducing cleavage efficiency in the RNase H and Cas9 enzymes used in the ASO and CRISPR techniques, respectively (11,12). To enhance the activity of RNase H and Cas9, different strategies have been applied (57–59) and the use of truncated ASO (antisense oligonucleotide) or sgRNA (single guide RNA) has been proven as an important technique for reducing off-target binding without changing on-target efficiency. Zhang *et al.* proposed *in vitro* pre-screening of sgRNA showing that truncated sgRNA enhanced target specificity and 16 mer sgRNA was found to be the most efficient one for the used target site (60). Different groups published *in vivo* studies where reducing the length of sgRNA from 20 to 17 nucleotides resulted in an improvement of target specificity for the *GFP* reporter gene, however, significant shortening of sgRNA (below 17 mer) can also cause unsatisfactory cleavage of target DNA in CRISPR-Cas9 technique (61,62). Similar studies have also been published in association with ASO drug design. The target specificity of ASO can be improved by shortening the length of modified ASO from 20 mer to 13 mer for targeting *Apolipoprotein B* (63). The length factor has been continuously considered as an important parameter to improve not only the on-target specificity but also cleavage efficiency. Lloyd *et al.* reported the cleavage efficacies of RNase H using oligonucleotides of different lengths (8–20 mer) upon *TNFα* mRNA target sites and a decreasing order of cleavage efficacy was found as 16 mer > 12 mer > 20 mer > 8 mer (64). Moreover, an extensive theoretical-experimental comparison study revealed a strong correlation between the structure and stability of hybrid duplexes and the cleavage efficiency of Cas9, which suggested that hybrid duplexes formed by 8–17 mer sgRNA provided a major contribution for the higher efficiency (65). Previous studies have clearly indicated that oligonucleotides of optimal length hybridize with the target site with optimum binding affinity that probably results in maximal specificity and efficiency (60,64,65). Although optimal length varies depending on the target site or other modifications, the optimal binding affinity could be the same. If more than one potential target sites have similar binding affinity, the most favorable target site should be the one that having minimum off target binding sites. The sequence of potential off target binding sites is also important, however the length shortening up to the optimal length was only developed here using the optimized binding affinity. Since free energy change (Δ*G*°_37_) is directly proportional to the logarithm of the binding affinity which is quantified by dissociation constant between the oligonucleotide and the target, the optimum value of Δ*G*°_37_ can give an indication of maximum cleavage efficiency and target specificity. A kinetic study also found supporting evidence for the hypothesis that the increase of cleavage efficiency of RNase H results from binding affinity rather than length (66). Therefore, prediction of Δ*G*°_37_ with significant accuracy for hybridization in physiological conditions is essential for drug design in oligonucleotide based therapeutics like ASO and CRISPR-Cas9.

Here we present a correlation between the Δ*G*°_37_ predicted by the derived parameters (Table 2) and their knockout efficiency from the previous reports (60–62,67) in Supplementary Table S7. At first, the predicted Δ*G*°_37_ for hybrid duplexes of truncated sgRNAs (17 mer) and their knockout efficiencies were compared (Supplementary Table S7). As a result, we found that not all the truncated sgRNAs (17 mer) have the maximal efficiency. However, the cleavage efficiencies of all 17 mer sgRNAs were highly correlated with the hybrid stabilities predicted by our parameters. For example, the sgRNA binding target sites with predicted Δ*G*°_37_ above −20 kcal mol^−1^ according to our parameters showed maximum knockout efficiency and the efficiency decreased with lowering the value of predicted Δ*G*°_37_ below −20 kcal mol^−1^. When the predicted Δ*G*°_37_ value remained between −18 and −20 kcal mol^−1^, the truncated sgRNA could even show significant efficiency. However, truncated sgRNA which can hybridize with less stability than −18 kcal mol^−1^ displayed a sudden decrease in efficiency. Thus, the stability predicted by our parameters can explain the differences in knockout efficiencies for sgRNA of the same length. We also included other sgRNA with different lengths (13–20 mer) studied by several groups (60–62,67) in the comparison (Supplementary Table S7). The comparison similarly supported the correlation between hybrid stabilities estimated by our parameters and the Cas9 cleavage efficiencies. Therefore, to design sgRNA of maximum specificity and efficiency, we optimized the predicted value of Δ*G*°_37_ in a physiological condition to be −20 kcal mol^−1^ using our parameters, whereas previous parameters (16) could not be used for this optimization. When the knockout efficiency of each sgRNA was compared with the stability (Δ*G*°_37_) in the physiological salt condition calculated using the old parameters applying the linear Equations (4) in Supplementary Table S7, significant correlation was not found. Initially, large differences were observed between the hybrid stabilities for each sgRNA predicted by old and new parameters in the physiological condition. As we demonstrated that the new parameters can better predict hybrid stability compared to the old ones in the physiological salt condition, the stability calculated by the new parameters can be considered more reliable. The predicted stabilities determined in physiological condition using old parameters were much lower which can mislead the assessment of optimal binding affinity of hybridization as well as optimal length of sgRNA. The hybrid stabilities predicted using old parameters cannot explain the phenomenon observed in the knockout experiments that the new parameters can, such as the differences seen in the knockout efficacies when applying truncated sgRNA (17 mer). Therefore, the new NN parameters will be more applicable not only for the precise prediction of hybrid stability in physiological salt condition but also to help in the design of oligonucleotide based therapeutics.

Moreover, an enhancement of binding affinity and hence also cleavage efficiency were observed as a result of using a purine rich sgRNA sequence which targeted a pyrimidine rich site (12), and the observation can be also explained by our NN parameters. In physiological condition, the rG−dC or rC−dG rich NN base pairs and also the NNs containing RNA with higher purine content are much more stabilized than others, which induce a higher binding affinity. Therefore, we can apply truncated sgRNA with much shorter length, thereby inducing higher specificity and efficiency. For example, the pyrimidine-rich DNA 20 mer replicates of the *MALAT1* oncogene (Supplementary Figure S11) were reported to be cleaved the fastest among all replicates with a changing sequence content according to the quantitative CRISPR PCR performed by Terrazas *et al.* (12). Herein, the stability (Δ*G*°_37_) of the corresponding hybrid duplex formation with the DNA 20 mer replicate was calculated using the old and new parameters simultaneously (Supplementary Figure S11). The predicted Δ*G*°_37_ was −20.1 kcal mol^−1^ according to the old parameters. However, the new parameters can estimate the stability with more accuracy and the predicted Δ*G*°_37_ obtained using the new parameters was −24.6 kcal mol^−1^, indicating a much higher stability than that calculated using old parameters applying the linear Equations (4) (Supplementary Figure S11). Therefore, to attain the optimum stability required for maximum cleavage efficiency and specificity in gene editing by Cas9, sgRNA length can be shortened from 20 mer to 16 mer for the target site (Supplementary Figure S11). Although continuous ‘G’-rich residue in sgRNA can be expected to hybridize with much higher stability, the efficiency was sometimes observed to be unexpectedly low (60–62,67). The formation of intramolecular stable conformations by ‘G’-rich oligomers could explain the exceptional correlation seen between the efficiency and the Δ*G*°_37_ predicted by our parameters. The precise prediction of hybrid stability in physiological condition can also be used to design truncated ASO having optimal binding affinity for any target site inducing higher specificity and efficiency of RNase H cleavage. However, modified DNA is generally used as ASO drugs to improve bio-stability and cellular uptake of the oligonucleotides. The stability of hybridization with ASO is reported higher than that with unmodified DNA (68,69). Therefore, truncated ASO with a much shorter length than the sgRNA can be used to obtain optimal binding affinity. A recent report clearly suggested that duplex stability of modified ASO can be correlated with the stability of unmodified duplex in 100 mM NaCl solution (68). The stability changes of hybrid duplexes in physiological salt concentration resulting from replacing DNA with different kinds of chemically-modified ASO were published earlier (68,69). Increases in melting temperature (Δ*T*_m_) per modification were reported for several modified ASO in comparison to unmodified DNA. Therefore, we can predict the stability (*T*_m_) of several RNA/ASO hybrids from the stability of RNA/DNA predicted by our new NN parameters in the physiological salt condition. The optimal stability predicted by our parameters can help in the selection of different target sites and in shortening the sgRNA and ASO up to the optimal length to improve target specificity and cleavage efficiency (70).

In summary, we derived a set of NN parameters to calculate hybrid stability in 100 mM NaCl buffer with significant accuracy. The effect of different cations like Na^+^, K^+^ and Mg^2+^ which are the major components occurring in physiological conditions on hybrid stability were investigated here. The applicability of our new parameters in predicting stability of hybrids even in 100 mM KCl and 100 mM NaCl in the presence of 1 mM Mg^2+^ was verified. Therefore, the improved parameters can be applied to predict stability of hybrid duplexes in different physiological conditions of extracellular and intracellular fluids. We also demonstrated that the derived NN parameters can be used in shortening of sgRNA and ASO up to an optimal length to enhance the target specificity and efficiency in CRISPR–Cas9 and antisense technique, respectively.

## Supplementary Material

gkaa572_Supplemental_File
