# Correction to ‘Improved nearest-neighbor parameters for the stability of RNA/DNA hybrids under a physiological condition’

**DOI:** 10.1093/nar/gkab780

**Published:** 2021-09-14

**Authors:** Dipanwita Banerjee, Hisae Tateishi-Karimata, Tatsuya Ohyama, Saptarshi Ghosh, Tamaki Endoh, Shuntaro Takahashi, Naoki Sugimoto

**Affiliations:** FIBER (Frontier Institute for Biomolecular Engineering Research), Konan University, 7-1-20 Minatojima-Minamimachi, Chuo-ku, Kobe 650-0047, Japan; FIBER (Frontier Institute for Biomolecular Engineering Research), Konan University, 7-1-20 Minatojima-Minamimachi, Chuo-ku, Kobe 650-0047, Japan; FIBER (Frontier Institute for Biomolecular Engineering Research), Konan University, 7-1-20 Minatojima-Minamimachi, Chuo-ku, Kobe 650-0047, Japan; FIBER (Frontier Institute for Biomolecular Engineering Research), Konan University, 7-1-20 Minatojima-Minamimachi, Chuo-ku, Kobe 650-0047, Japan; FIBER (Frontier Institute for Biomolecular Engineering Research), Konan University, 7-1-20 Minatojima-Minamimachi, Chuo-ku, Kobe 650-0047, Japan; FIBER (Frontier Institute for Biomolecular Engineering Research), Konan University, 7-1-20 Minatojima-Minamimachi, Chuo-ku, Kobe 650-0047, Japan; FIBER (Frontier Institute for Biomolecular Engineering Research), Konan University, 7-1-20 Minatojima-Minamimachi, Chuo-ku, Kobe 650-0047, Japan; FIRST (Graduate School of Frontiers of Innovative Research in Science and Technology), Konan University, 7-1-20 Minatojima-Minamimachi, Chuo-ku, Kobe 650-0047, Japan

The authors wish to make the following corrections to their article (1).

**Correction 1: Tables 1 and 2**

At the time of Δ*S*° calculation in Table 2, we used *ΔH*° and *ΔG*°_37_ values up to three decimal places. However, we showed the values with one decimal place in Table 2. Using the values of *ΔH*° and *ΔS*° in Table 2, we have recently noticed that some values of *ΔG*°_37_ calculated using the Gibbs free energy relation are not accurate due to the rounding of the numbers. Therefore, we revised all the *ΔS*° parameters of Table 2 by recalculating them using the Gibbs free energy relation and *ΔG*°_37_ and *ΔH*° values up to one decimal place. Since the nearest-neighbor parameters were corrected for *ΔS*° in Table 2, the predicted values in Table 1 were also marginally modified. The modified parts are highlighted in **bold** in the new Tables 1 and 2 provided below. Due to the correction of the predicted values in Table 1, the average differences for the measured and predicted *ΔG*°_37_ and *T*_m_ should also be revised in the main text as shown in bold below:

- Page 12042.

Predicted Δ*G*°_37_ and *T*_m_ values based on the established NN parameters agreed well with the measured values with **3.0%** and **1.2°C** deviations, respectively.

- Page 12043.

To improve the prediction, we developed new NN parameters that significantly reduced the average differences for the measured and predicted *ΔG*°_37_ and *T*_m_ (**3.0%** and **1.2°C**, respectively).

- Page 12049.

Our parameters were able to predict the stability of designed 38 RNA/DNA hybrids (Table 1) adequately, with average values of prediction error for *ΔH*°, *ΔS*° and *ΔG*°_37_ and of 9.0%, 10.1% and **3.0%**, respectively, and for *T*_m_ of only **1.2°C**.

**Table 1.** Thermodynamic parameters of RNA/DNA hybrid duplexes in 100 mM NaCl buffer solution

No.	RNA sequences^a^	Measured parameters in 100 mM NaCl solution^b^				Old prediction^d^		New prediction^e^	
		Δ*H*° /kcal mol^−1^	*T*Δ*S*° /kcal mol^−1^	Δ*G*°_37_ /kcal mol^−1^	*T*_m_^c^ /°C	Δ*G*°_37_ /kcal mol^−1^	*T*_m_^c^ /°C	Δ*G*°_37_ /kcal mol^−1^	*T*_m_^c^ /°C
1	GGUCGC	−42.6 ± 1.5	−36.0 ± 1.3	−6.6 ± 0.3	27.2	−6.0	**20.6**	−6.5	**27.3**
2	CGGACC	−55.0 ± 2.3	−48.6 ± 2.1	−6.4 ± 0.4	26.1	−6.1	**21.2**	−6.4	27.1
3a	GCCGUGAG	−72.5 ± 2.4	−63.4 ± 2.1	−9.1 ± 0.4	41.2	−7.5	**32.0**	−8.9	**41.1**
3b	GAGCCGUG	−78.1 ± 2.5	−69.1 ± 2.3	−9.0 ± 0.4	41.5	−7.5	**32.0**	−8.9	**41.1**
4a	GUCAGACU	−57.1 ± 1.4	−50.4 ± 1.2	−6.7 ± 0.2	29.7	−5.8	**19.0**	−6.7	30.1
4b	GACAGUCU	−54.2 ± 2.9	−47.3 ± 2.5	−6.9 ± 0.5	30.1	−5.8	**19.0**	−6.7	30.1
5	GAACUGCC	−66.7 ± 1.8	−59.4 ± 1.7	−7.3 ± 0.3	33.5	−7.1	**29.9**	−7.4	**33.8**
6	GGCAGUUC	−78.2 ± 3.7	−71.0 ± 3.4	−7.2 ± 0.5	33.8	−6.7	27.0	−7.4	34.0
7	GCGAUCGGA	−77.6 ± 2.8	−67.9 ± 2.4	−9.7 ± 0.5	43.5	−8.5	**37.2**	−9.9	45.2
8	GCCAGUAGG	−74.8 ± 2.3	−65.4 ± 2.0	−9.4 ± 0.4	42.6	−8.5	38.2	−9.7	43.7
9	GUUCAAUACG	−62.9 ± 2.3	−57.0 ± 2.1	−5.9 ± 0.3	27.5	−6.0	**23.3**	−6.3	29.6
10	AGGAUGACCG	−79.1 ± 1.5	−68.7 ± 1.3	−10.4 ± 0.3	45.9	−9.6	**43.2**	**−11.2**	**49.2**
11	CGCUUGUUAC	−76.9 ± 2.4	−69.9 ± 2.2	−7.0 ± 0.3	33.1	−6.7	**26.9**	−6.4	30.3
12	GUAACAAGCG	−81.6 ± 2.2	−72.9 ± 1.9	−8.7 ± 0.3	39.2	−7.8	**33.4**	−8.7	39.5
13	CACUUGUUAC	−73.9 ± 1.6	−68.0 ± 1.5	−5.9 ± 0.2	28.1	−5.8	**21.9**	−5.7	27.6
14a	AAUCUGGCCA	−57.8 ± 2.7	−48.7 ± 2.3	−9.1 ± 0.5	42.8	−8.8	**39.0**	−9.1	41.2
14b	AUGGCUCCAA	−64.5 ± 2.6	−55.6 ± 2.2	−8.9 ± 0. 4	40.1	−8.8	**39.0**	−9.1	41.2
15	GGGGAACAAGG	−110.5 ± 2.4	−96.6 ± 2.1	−13.9 ± 0.4	54.3	−12.1	**54.4**	−14.1	**55.5**
16	UUCACCUGGUC	−85.1 ± 1.9	−74.8 ± 1.7	−10.3 ± 0.3	45.3	−9.0	**38.7**	**−10.7**	**46.8**
17a	GGCAGGAAUCCG	−100.8 ± 3.0	−86.6 ± 2.6	−14.2 ± 0.5	56.8	−12.1	**51.1**	−14.2	**56.6**
17b	GGAAUCAGGCCG	−107.5 ± 4.3	−93.1 ± 3.8	−14.4 ± 0.7	56.3	−12.1	**51.1**	−14.2	**56.6**
18a	UAUCUUCCGAAU	−60.7 ± 1.7	−54.0 ± 1.5	−6.7 ± 0.3	30.2	−7.7	**31.2**	−7.0	32.9
18b	UAUCCUUCGAAU	−57.5 ± 1.2	−50.9 ± 1.1	−6.6 ± 0.2	29.6	−7.7	**31.2**	−7.0	32.9
19a	AAUGGAUUACAA	−83.1 ± 2.2	−75.3 ± 2.0	−7.8 ± 0.3	36.3	−8.2	33.9	−8.0	**36.7**
19b	AUUGGAUACAAA	−79.3 ± 3.6	−71.3 ± 3.2	−8.0 ± 0.5	36.2	−8.2	33.9	−8.0	**36.7**
20	CCUGGAAUCCAA	−85.1 ± 2.4	−74.0 ± 2.1	−11.1 ± 0.4	48.2	−9.9	**42.6**	−11.6	**49.3**
21	GGCUCAAUUGAC	−100.4 ± 2.0	−89.7 ± 1.8	−10.7 ± 0.3	45.2	−9.8	**42.0**	−10.8	**46.3**
22a	CGGCCUUGAUCC	−104.8 ± 4.0	−92.1 ± 3.5	−12.7 ± 0.6	51.9	−11.0	**45.8**	−12.2	**51.2**
22b	CGGAUUCCUGCC	−92.4 ± 2.6	−80.5 ± 2.3	−11.9 ± 0.4	50.3	−11.0	**45.8**	−12.2	**51.2**
23	UCCGAAUUAUCU	−81.5 ± 2.8	−73.6 ± 2.6	−7.9 ± 0.4	35.8	−7.7	**31.2**	−7.0	32.9
24	AGAUAAUUCGGA	−83.5 ± 2.4	−75.8 ± 2.2	−7.7 ± 0.3	35.5	−8.6	35.5	−8.6	**38.8**
25	GCUUCUCUCUUC	−73.1 ± 1.1	−66.4 ± 1.0	−6.7 ± 0.1	31.5	−7.7	**31.7**	−6.8	**32.0**
26	GAAGAGAGAAGC	−84.8 ± 4.1	−72.3 ± 3.5	−12.5 ± 0.7	54.0	−10.5	**49.3**	−12.6	52.0
27	UCGUUCUUGUCU	−77.8 ± 2.1	−70.0 ± 1.9	−7.8 ± 0.3	36.4	−7.4	**29.9**	−7.8	36.0
28	AGACAAGAACGA	−95.9 ± 2.1	−84.7 ± 1.8	−11.2 ± 0.3	47.6	−10.0	**44.7**	−11.5	48.1
29a	GUUAGCGUUACGC	−89.2 ± 3.1	−78.8 ± 2.7	−10.4 ± 0.5	45.0	−10.1	**40.3**	−11.0	45.9
29b	GCGUUUACGUAGC	−102.6 ± 1.1	−91.1 ± 1.0	−11.5 ± 0.2	47.8	−10.1	**40.3**	−11.0	45.9
30	UCACGUAGUCGUAU	−113.6 ± 4.4	−101.0 ± 3.9	−12.6 ± 0.7	49.8	−10.2	**40.1**	−12.6	**50.6**

^a^The designed hybrid duplex consists of a denoted RNA strand and its complementary DNA strand; the denoted RNA strand is represented as GCCGUGAG for 5’rGCCGUGAG3’. The pair of hybrid duplexes with identical nearest-neighbors are given by the number Xa and Xb (X is 3, 4, 14, 17, 18, 19, 22 and 29).

^b^All experiments were carried out in solutions containing 100 mM NaCl, 10 mM Na_2_HPO_4_, and 1 mM Na_2_EDTA (pH 7.0).

^c^The melting temperatures were determined at a total oligomer strand concentration of 8 μM.

^d^The hybrid stability in 100 mM NaCl buffer was predicted using Equations (4) and (5) (21) at the respective stability in 1 M NaCl buffer, determined using NN parameters (16).

^e^The hybrid stability in 100 mM NaCl buffer was predicted using new nearest-neighbor parameters (Table 2). The average prediction error *ΔΔG*°_37_ (measured *ΔG*°_37_ − predicted *ΔG*°_37_) which is represented as the percentage of error with respect to predicted *ΔG*°_37_ and Δ*T*_m_ (measured *T*_m_ – predicted *T*_m_) were calculated for old prediction as 10.7% and **5.0°C**, respectively and new prediction as **3.0%** and **1.2°C**, respectively.

**Table 2.** Nearest-neighbor parameters of hybrid duplex in 100 mM NaCl buffer solution^a^

Sequence	Δ*H*°_NN_ /kcal mol^−1^	Δ*S*°_NN_ /cal mol^−1^K^−1^	Δ*G*°_37NN_ /kcal mol^−1^
rAA/dTT	−7.8	−22.9	−0.7
rAC/dGT	−10.1	**−27.7**	−1.5
rAG/dCT	−9.4	**−26.1**	−1.3
rAU/dAT	−5.8	**−17.4**	−0.4
rCA/dTG	−9.8	**−27.7**	−1.2
rCC/dGG	−9.5	**−25.1**	−1.7
rCG/dCG	−9.0	**−24.5**	−1.4
rCU/dAG	−6.1	**−18.4**	−0.4
rGA/dTC	−8.6	**−22.9**	−1.5
rGC/dGC	−10.6	−27.7	−2.0
rGG/dCC	−13.3	**−35.5**	−2.3
rGU/dAC	−9.3	−25.5	−1.4
rUA/dTA	−6.6	−19.7	−0.5
rUC/dGA	−6.5	**−16.4**	−1.4
rUG/dCA	−8.9	**−23.5**	−1.6
rUU/dAA	−7.4	**−24.5**	0.2
init. rG − dC/rC − dG^b^	0	**−6.4**	2.0
init. rA − dT/rU − dA^c^	0	**−8.4**	2.6

^a^16 nearest-neighbor parameters and initiation for rG − dC or rC − dG pairing (init. rG − dC/rC − dG) and initiation for rA − dT or rU − dA pairing (init. rA − dT/rU − dA) derived for RNA/DNA hybrid in 100 mM NaCl solution using the data of Table 1. The average errors estimated for Δ*H*°_NN_, Δ*S*°_NN_, and Δ*G*°_37NN_ in 100 mM NaCl solution were ± 0.08 kcal mol^−1^, ±0.35 cal mol^−1^ K^−1^ and ± 0.06 kcal mol^−1^, respectively.

^b^Initiation parameters for the duplexes that contain at least one rG − dC or rC − dG base pair in any terminal.

^c^Initiation parameters for the duplexes that contain only rA − dT or rU − dA base pairs in both terminals.

**Correction 2: Tables S1, S3, S4 and S5**

We found that several parameters of *T*_m_ using some data in Table 1 were calculated with the value of *R* (gas constant) as 0.00198 kcal mol^−1^ K^−1^ due to an error in the calculation algorithm. We recalculated the value of *R* as 0.001987 kcal mol^−1^ K^−1^ and revised Tables S1, S3, S4 and S5. Due to the correction of Table S5, the average differences for the measured and predicted *ΔG*°_37_ and *T*_m_ should also be revised in the main text as shown below:

- Page 12050.

A comparison (Supplementary Table S5) of the measured stabilities of the hybrid duplexes in 100 mM KCl and the corresponding stabilities predicted using the new parameters clearly displayed that the new parameters (Table 2) can predict the stability (*ΔG*°_37_ and *T*_m_) of hybrid duplexes in physiological conditions even in the presence of K^+^ ions with average prediction errors of **7.9%** and **2.8°C**, respectively.

**Correction 3: Storage server**

To calculate the thermodynamic parameters (*ΔH*°, *ΔS*° and *ΔG*°_37_) of a hybrid duplex in 100 mM NaCl solution using derived new NN parameters, an open access website was created. However, the server that was storing the data is no longer available for the convenience of the provider.

Thus, we would like to update the storage server to the following and revise a sentence in the main text and in Supporting Information on page 4.

http://www.konan-fiber.jp/hp/sugimoto/contents/NN/NearestNeighborCalculator.htm

- Page 12049.

To reduce the effort for calculating hybrid stability in 100 mM NaCl condition by our parameters (Table 2), we designed the website located at http://www.konan-fiber.jp/hp/sugimoto/contents/NN/NearestNeighborCalculator.htm.

- Supporting Information on page 4

To calculate the thermodynamic parameters (*ΔH*°, *ΔS*°, and *ΔG*°_37_) of a hybrid duplex in 100 mM NaCl solution using derived new NN parameters, an open access website was created which is located at http://www.konan-fiber.jp/hp/sugimoto/contents/NN/NearestNeighborCalculator.htm.

These changes do not affect the results, discussion and conclusions presented in the article.

## Supplementary Material

gkab780_Supplemental_File
