# Supplementary material for: Improved nearest-neighbor parameters for the stability of RNA/DNA hybrids under a physiological condition
Source: Nucleic Acids Res. 2020 Jul 14;48(21):12042–54. doi: 10.1093/nar/gkaa572 (PMC7708073; doi:10.1093/nar/gkaa572)
Supplement: gkaa572_Supplemental_File [file gkaa572_supplemental_file.pdf]

Supporting Information

Improved nearest-neighbor parameters for the stability of RNA/DNA hybrids under a physiological condition

Dipanwita Banerjee[1], Hisae Tateishi-Karimata[1], Tatsuya Ohyama[1], Saptarshi Ghosh[1], Tamaki Endoh[1], Shuntaro Takahashi[1], and Naoki Sugimoto*[1, 2]

[1]FIBER (Frontier Institute for Biomolecular Engineering Research), Konan University, 7-1-20 Minatojima-Minamimachi, Chuo-ku, Kobe 650-0047, Japan, [2]FIRST (Graduate School of Frontiers of Innovative Research in Science and Technology), Konan University, 7-1-20 Minatojima-Minamimachi, Chuo-ku, Kobe 650-0047, Japan

List of contents:

1. Method of nearest-neighbor parameter calculation.

2. **Table S**1: Thermodynamic stabilities measured and predicted for hybrid duplexes in 1 M NaCl solution.

3. **Table S**2: Measured stabilities in 100 mM and predicted stabilities in 1 M NaCl solution for hybrid duplexes with respective sequence factors.

4. **Table S**3: Nearest-neighbor parameters for RNA/DNA hybrids in 1 M NaCl solution.

5. **Table S**4: Measured and predicted stabilities of hybrid duplexes in buffer solution containing 100 mM NaCl.

6. **Table S**5: Thermodynamic parameters measured and predicted for RNA/DNA hybrids in 100 mM KCl buffer solution.

7. **Table S**6: Thermodynamic parameters measured for RNA/DNA hybrids in 100 mM NaCl buffer solution with and without 1 mM $MgCl_2$.

8. **Table S**7: Comparison of sgRNA binding affinity based on stability predicted using old and new parameters with cleavage efficiency.

9. **Figure S**1: MS and HPLC of oligonucleotides corresponding to several model hybrid sequences.

10. **Figure S**2: Temperature induced unfolding CD assays and UV-melting curves of denaturation and renaturation for several model hybrid sequences.

11. **Figure S**3: CD spectra of respective RNA/DNA hybrids selected from Table 1 in buffer solutions containing 1 M and 100 mM NaCl using 20 μM of total strand concentration at 4 °C.

12. **Figure S**4: Plot of melting temperatures of selected hybrid duplexes in different sodium ion concentration versus log [Na^+].

13. **Figure S**5: Effect of sequence factors f(G–C) and f(rPu) on the hybrid stabilities ($\Delta G°_{37}/N_{total}$) in 100 mM NaCl solution.

14. **Figure S**6: The measured $\Delta G°_{37}$ in 100 mM NaCl versus predicted $\Delta G°_{37}$ in 1 M NaCl for all hybrid sequences in one plot.

15. **Figure S**7: Normalized UV melting curves and T_m^{-1} versus ln (C_t/4) plots of representative hybrid duplex pairs 3a and 3b having identical nearest-neighbor.

16. **Figure S**8: The plot of measured $\Delta G°_{37}$ and T_m in 100 mM NaCl vs that in 100 mM KCl using the data of four

sequences of Table S5.

17. **Figure S**9: Normalized UV melting curves in a physiological salt solution, 1 M NaCl, and 100 mM NaCl solution of hybrid duplexes from Group A, Group C, and Group B, respectively.

18. **Figure S**10: The plot of measured $\Delta G°_{37}$ (A) and T_m (B) in presence versus in absence of 1 mM $MgCl_2$ in a buffer containing 100 mM NaCl for the three selected three sequences from Table S6 belonging to three different groups (Group A, Group B, and Group C).

19. **Figure S**11: Schematic presentation about the advantage of the precise prediction for hybrid duplex in CRISPR-Cas9 gene-editing technique.

Calculation of nearest-neighbor parameters

According to the nearest-neighbor (NN) model, the free energy change of the duplex formation consists of two terms: (i) Free energy change for helix propagation as the sum of each subsequent base pair present in the sequence. (ii) Free energy change for helix initiation to form a first base pair in the double helix. Since there are two possibilities for helix initiation of RNA/DNA hybrid, i.e., either by rG–dC/rC–dG pairing or rA–dT/rU–dA pairing, two initiation factors are considered here. The 16 NN base pairs and two initiation factors were determined using the thermodynamic data for 38 RNA/DNA duplexes in 100 mM NaCl buffer solution, which were obtained from the UV melting experiment and a non-linear least square computer program written in Python. The program calculated $\Delta G°_{37}$ using parameter sets containing 18 parameters (16 Watson-Crick NN base pairs, and 2 terminal pairs). The algorithm for determining the parameters was as follows. First, the initial parameter set (p) with 18 parameter values were assumed. Based on p, positive (p+) and negative (p−) parameter sets were prepared. The p+ and p− had a larger or smaller parameter, respectively, determined by the specific small value (ΔE) compared to p. Further, the ΔE was set to 0.1. For example, the positive parameter set for rAA/dTT, $p+_{AA/TT}$, was written by (rAA/dTT, rAC/dGT, rAG/dCT, rAU/dAT, ...) = (0.1, 0.0, 0.0, 0.0, ...). Since p, p+, and p− were prepared for each parameter, the total number of parameter sets was 54. The $\Delta G°_{37}$ value for each sequence was calculated using these parameter sets and compared with the error sums of squares for $\Delta G°_{37}$ between the experimental and predicted ($\Delta\Delta G°_{37}$) ($\Delta\Delta G°_{37} = \sum(E_{exp.} - E_{predict})^2$). As a result, the parameter set that minimized $\Delta\Delta G°$ was adopted as the next parameter set. Based on the determined parameter set, new positive and negative parameter sets were prepared, and calculations for $\Delta G°$ carried out using these parameter sets. These calculations were repeated until $\Delta\Delta G°$ is unchanged. When $\Delta\Delta G°$ did not change, $\Delta G°$ for each sequence was calculated using parameter sets with half specific small value ($\Delta E/2$). These procedures were repeated until ΔE was < 0.001. The calculation for $\Delta H°$ is the same as the procedure mentioned above. Nearest-neighbor parameters for $\Delta H°$ were determined in the same way as for $\Delta G°_{37}$ and the initiation values of $\Delta H°$ were fixed as zero to minimize the error of prediction for other $\Delta H°$ values of 16 nearest-neighbors. The $\Delta S°$ values were obtained from the determined values of $\Delta G°_{37}$ and $\Delta H°$ using equation 2 mentioned in method section for determining of thermodynamic parameters for RNA/DNA hybrids. To check the accuracy and reliability of the developed program we calculated the NN parameters for RNA/DNA hybrids in 1 M NaCl solution with new program, and compared with the reported parameters (Table S3) (1). We have found that new algorithm can predict the stabilities of reported 64 RNA/DNA hybrids better than previous parameters can do (1). The average prediction errors for $\Delta H°$, $\Delta S°$ and $\Delta G°_{37}$ of reported 64 hybrid sequences are 6%, 6.9%, and 3.8%, respectively using derived parameters by new algorithm (Table S3), whereas, 6.8%, 7.6%, and 5.4%, respectively using reported parameters in 1 M NaCl solution (1). The average differences between the NN parameters calculated by our program and the reported one were ±1.7 kcal mol^{-1}, ±5.4 cal mol^{-1} K^{-1}, and ±0.2 kcal mol^{-1} for $\Delta H°$, $\Delta S°$, and $\Delta G°_{37}$, respectively, suggesting that this improved program can be applied for more precise determination of the NN parameters for RNA/DNA hybrids in 100 mM NaCl buffer solution.

To calculate the thermodynamic parameters ($\Delta H°$, $\Delta S°$, and $\Delta G°_{37}$) of hybrid duplex in 100 mM NaCl solution using derived new NN parameters, an open access website was created which is located at https://drive.google.com/open?id=1xF0TianMpQ6rwszN9gsweu7C5Wnzq0kF. The program was implemented in

this website by javascript. In this work, the comparison between the hybrid stability in 100 mM NaCl solution and in 1 M NaCl solution is needed. We may also need to compare the stability of hybrid duplex with that of DNA and RNA duplexes having equivalent base composition. Therefore, we inserted NN parameters of DNA, RNA, and RNA/DNA hybrid duplexes in 1 M NaCl solution along with our parameters derived here. To evaluate the thermodynamic parameters of hybrid duplex using the website, we have to input the sequence and salt concentration. The website can automatically determine the constituting NN parameters and calculate the thermodynamic parameters. There is a provision in the website to calculate duplex stability using other set of reported NN parameters by manually applying the parameters for convenience.

Table S1. Thermodynamic stabilities measured and predicted for hybrid duplexes in 1 M NaCl solution

No.	RNA Sequence[a]	Measured[b] in 1 M NaCl		Predicted[d] in 1 M NaCl		Prediction errors[e]	
		ΔG°_{37} /kcal mol^{-1}	T_m[c] /°C	ΔG°_{37} /kcal mol^{-1}	T_m[c] /°C	$\Delta\Delta G^\circ_{37}$/kcal mol^{-1}	ΔT_m/°C
1a	GCCGUGAG	−10.0	46.6	−9.2	42.6	−0.8 (9 %)	4.0
3	GAACUGCC	−8.7	39.9	−8.6	40.3	−0.1 (1 %)	−0.4
6	GCCAGUAGG	−10.8	49.7	−10.8	49.5	−0.0 (0 %)	0.2
7	GUUCAAUACG	−7.6	35.3	−6.9	32.6	−0.7 (10 %)	2.7
8	AGGAUGACCG	−12.9	54.9	−12.6	55.4	−0.3 (2 %)	0.5
9	CGCUUGUUAC	−8.6	39.4	−8.0	36.8	−0.6 (7 %)	2.6
17a	AAUGGAUUACAA	−10.6	45.8	−10.3	44.6	−0.3 (3 %)	1.2

[a]Seven RNA sequences were selected from Table 1. [b]All experiments were done in the buffer solution of 1 M NaCl, 10 mM Na_2HPO_4, and 1 mM Na_2EDTA (pH 7.0). [c]Melting temperatures are given at the total strand concentration of 8 μM. [d]Stabilities of hybrid duplex in 1 M NaCl buffer solution were predicted using previous nearest neighbor parameters (1). [e]Prediction errors $\Delta\Delta G^\circ_{37}$ and ΔT_m were calculated same as Table 1. The average value of $\Delta\Delta G^\circ_{37}$ and ΔT_m are obtained 4.6% and 1.7 °C, respectively.

Table S2. Measured stabilities in 100 mM and predicted stabilities in 1 M NaCl solution for hybrid duplexes with respective sequence factors

Sequence[a]	f(G–C)[b]	f(rPu)[c]	Group[d]	Measured $\Delta G°_{37}$ (100 mM)[e] /kcal mol^{-1}	Predicted $\Delta G°_{37}$ (1 M)[f] /kcal mol^{-1}
GCCGUGAG[g]	0.5	0.3	A	−9.1	−9.2
GAGCCGUG[g]	0.5	0.3	A	−9.0	−9.2
GUCAGACU[g]	0	0	C	−6.7	−6.5
GACAGUCU[g]	0	0	C	−6.9	−6.5
GAACUGCC[g]	0.3	0	C	−7.3	−8.6
GGCAGUUC[g]	0.3	0	C	−7.2	−8.0
GCGAUCGGA[g]	0.3	0.3	A	−9.7	−10.9
GCCAGUAGG[g]	0.3	0.3	A	−9.4	−10.8
GUUCAAUACG[g]	−0.2	0	C	−5.9	−6.9
AGGAUGACCG[g]	0.2	0.4	A	−10.4	−12.6
CGCUUGUUAC[g]	0	−0.4	C	−7.0	−8.0
GUAACAAGCG[g]	0	0.4	C	−8.7	−9.8
CACUUGUUAC[g]	−0.2	−0.4	B	−5.9	−6.6
AAUCUGGCCA[g]	0	0	C	−9.1	−11.4
AUGGCUCCAA[g]	0	0	C	−8.9	−11.4
GGGGAACAAGG[g]	0.3	0.8	A	−13.9	−16.6
UUCACCUGGUC[g]	0.1	−0.5	C	−10.3	−11.7
GGCAGGAAUCCG[g]	0.3	0.3	A	−14.2	−16.6
GGAAUCAGGCCG[g]	0.3	0.3	A	−14.4	−16.6
UAUCUUCCGAAU[g]	−0.3	−0.3	B	−6.7	−9.5
UAUCCUUCGAAU[g]	−0.3	−0.3	B	−6.6	−9.5
AAUGGAUUACAA[g]	−0.5	0.3	C	−7.8	−10.3
AUUGGAUACAAA[g]	−0.5	0.3	C	−8.0	−10.3
CCUGGAAUCCAA[g]	0	0	C	−11.1	−13.1
GGCUCAAUUGAC[g]	0	0	C	−10.7	−12.9
CGGAUUCCUGCC[g]	0.3	−0.3	C	-11.9	-14.8
GCGUUUACGUAGC[g]	0.1	−0.1	C	−11.5	−13.4
UCACGUAGUCGUAU[g]	−0.1	−0.1	B	−12.6	−13.5
CUACGCUU[g]	0	−0.5	C	−6.2	−6.0
AUUCGGAAGAUA[g]	−0.3	0.3	C	−9.8	−11.0
ACCGCA[h]	0.3	0	C	−6.4	−6.4

GCCAGUUAA[h]	−0.1	0	C	−6.9	−7.3
AUUGGAUACAAA[h]	−0.5	0.3	C	−7.8	−10.3
GCUUCUCUUC[i]	0	−0.8	C	−5.5	−7.2
GAAGAGAAGC[i]	0	0.8	C	−10.6	−10.9
UCCCUCCUCUCC[j]	0.3	−1.0	C	−9.6	−14.0
GGAGAGGAGGGA[j]	0.3	1.0	A	−15.6	−19.1
CCUUCCCUU[j]	0.1	−1.0	C	−5.8	−6.9
AAGGGAAGG[j]	0.1	1.0	A	−10.0	−12.5
UUCCCUUCC[j]	0.1	−1.0	C	−5.1	−7.5
GGAAGGGAA[j]	0.1	1.0	A	−10.1	−12.0
GCUCUCUGGC[j]	0.4	−0.4	C	−8.9	−12.5
GCCAGAGAGC[j]	0.4	0.4	A	−11.2	−13.3
CUCGUACCUUCCGGUCC[j]	0.3	−0.5	C	−12.7	−20.9
GGACCGGAAGGUACGAG[j]	0.3	0.3	A	−17.0	−25.5
CUCGUACCUUUCCGGUCC[j]	0.2	−0.6	C	−14.6	−21.1
GGACCGGAAAGGUACGAG[j]	0.2	0.6	A	−18.1	−26.5
UAGUUAUCUCUAUCU[j]	−0.5	−0.5	B	−7.5	−10.8
AGAUAGAGAUAACUA[j]	−0.5	0.5	C	−10.5	−13.8
GCACAGCC[j]	0.5	0	C	−7.9	−11.9
GGCUGUGC[j]	0.5	0	C	−8.1	−10.4
GAGCUCCCAGGC[j]	0.5	0	C	−13.4	−17.6
GCCUGGGAGCUC[j]	0.5	0	C	−14.3	−18.2
UGUACGUCACAACUA[j]	−0.2	−0.1	B	−11.2	−15.1
UAGUUGUGACGUACA[j]	−0.2	0.1	C	−11.8	−14.7
UAUACAAGUUAUCUA[j]	−0.6	−0.1	B	−7.8	−10.6
UAGAUAACUUGUAUA[j]	−0.6	−0.1	B	−7.7	−11.1
CGACUAUGCAAAAAC[j]	−0.2	0.2	C	−11.3	−15.7
GUUUUUGCAUAGUCG[j]	−0.2	−0.2	B	−8.7	−12.5
CGCAAAAAAAAAACGC[j]	−0.3	0.5	C	−13.0	−17.7
GCGUUUUUUUUUUGCG[j]	−0.3	−0.5	B	−5.9	−10.2

[a] Hybrid sequences from Table 1 and previous studies (2–4). [b] f(G–C) = ($N_{G–C}$ −$N_{A–U}$)/N_{total}, [c] f(rPu) = (N_{pu} −N_{py})/N_{total}. [d] A sequence was categorized in Group A when both f(G–C) and f(rPu) were positive, in Group B when both f(G–C) and f(rPu) were negative, and in Group C when f(G–C) was positive and f(rPu) was negative or f(G–C) was negative and f(rPu) was positive or any of f(G–C) and f(rPu) or both were zero. [e] Measured stabilities of all hybrid duplexes were obtained in a 100 mM NaCl-phosphate buffer solution (pH 7.0). [f] Predicted stabilities of hybrid duplexes were derived by the nearest-neighbor parameters in a 1 M NaCl solution (1). [g] Sequences were taken from Table 1. [h] Sequences collected from Nakano et al. (2). [i] Sequences taken from the report by Gyi et al. (3). [j] Sequences selected from the report by Lesnik and Freier (4).

Table S3. Nearest-neighbor parameters for RNA/DNA hybrids in 1 M NaCl solution

sequence	Parameters calculated by new program[a]			Parameters reported previously[b]		
	ΔH°_{NN} /kcal mol^{-1}	ΔS°_{NN} /cal mol^{-1}K^{-1}	ΔG°_{37NN} /kcal mol^{-1}	ΔH°_{NN} /kcal mol^{-1}	ΔS°_{NN} /cal mol^{-1}K^{-1}	ΔG°_{37NN} /kcal mol^{-1}
rAA/dTT	−7.5	−20.9	−1.0	−7.8	−20.8	−1.0
rAC/dGT	−8.5	−21.6	−1.8	−5.9	−12.3	−2.1
rAG/dCT	−8.6	−22.6	−1.6	−9.1	−23.5	−1.8
rAU/dAT	−7.5	−21.8	−0.7	−8.3	−23.9	−0.9
rCA/dTG	−8.4	−22.8	−1.3	−9.0	−26.1	−0.9
rCC/dGG	−7.9	−19.0	−2.0	−9.3	−23.2	−2.1
rCG/dCG	−14.8	−41.8	−1.9	−16.3	−47.1	−1.7
rCU/dAG	−4.4	−10.7	−1.1	−7.0	−19.7	−0.9
rGA/dTC	−9.4	−24.4	−1.8	−5.5	−13.5	−1.3
rGC/dGC	−10.5	−25.3	−2.6	−8.0	−17.1	−2.7
rGG/dCC	−12.5	−31.6	−2.7	−12.8	−31.9	−2.9
rGU/dAC	−9.5	−26.0	−1.5	−7.8	−21.6	−1.1
rUA/dTA	−9.5	−28.2	−0.7	−7.8	−23.2	−0.6
rUC/dGA	−8.1	−22.3	−1.2	−8.6	−22.9	−1.5
rUG/dCA	−8.6	−23.2	−1.4	−10.4	−28.4	−1.6
rUU/dAA	−5.6	−17.0	−0.4	−11.5	−36.4	−0.2
initiation	1.9	−3.9	3.1	1.9	−3.9	3.1

[a]Nearest-neighbor parameters calculated by new program using the same data reported previously (1). [b]Nearest-neighbor parameters proposed previously (1).

Table S4. Measured and predicted stabilities of hybrid duplexes in a buffer solution containing 100 mM NaCl[a]

Sequence[a]	Measured[b]		Predicted[d]		Prediction errors	
	$\Delta G°_{37}$/kcal mol^{-1}	T_m[c] /°C	$\Delta G°_{37}$/kcal mol^{-1}	T_m[c]/°C	$\Delta\Delta G°_{37}$[e]/kcal mol^{-1}	ΔT_m[f]/°C
GCCAGUUAA[g]	−6.9	30.6	−6.6	30.7	−0.3	−0.1
AUUGGAUACAAA[g]	−7.8	35.5	−8.0	36.8	0.2	−1.3
GCUUCUCUUC[h]	−5.5	23.7	−5.0	22.7	−0.5	1.0
GAAGAGAAGC[h]	−10.6	46.9	−9.8	43.9	−0.8	3.0
UCCCUCCUCUCC[i]	−9.6	43.4	−11.6	50.9	2.0	−7.5
GGAGAGGAGGGA[i]	−15.6	61.4	−17.1	63.3	1.5	−1.9
CCUUCCCUU[i]	−5.8	20.5	−4.9	22.0	−0.9	−1.5
AAGGGAAGG[i]	−10.0	44.8	−10.4	46.0	0.4	1.2
UUCCCUUCC[i]	−5.1	14.9	−5.9	26.6	0.8	−11.7
GGAAGGGAA[i]	−10.1	44.2	−10.6	46.9	0.5	−2.7
GCUCUCUGGC[i]	−8.9	40.8	−9.9	44.8	1.0	−4.0
GCCAGAGAGC[i]	−11.2	50.9	−11.8	51.1	0.6	−0.2
UAGUUAUCUCUAUCU[i]	−7.5	34.9	−7.6	35.5	0.1	−0.6
AGAUAGAGAUAACUA[i]	−10.5	45.4	−10.7	44.7	0.2	0.7
GCACAGCC[i]	−7.9	35.6	−8.9	40.8	1.0	−5.2
GGCUGUGC[i]	−8.1	37.2	−9.3	42.8	1.2	−5.6
GAGCUCCCAGGC[i]	−13.4	56.7	−14.8	58.7	1.4	−2.0
GCCUGGGAGCUC[i]	−14.3	60.3	−14.9	59.1	0.6	1.2
UGUACGUCACAACUA[i]	−11.2	49.2	−13.6	52.0	2.4	−2.8
UAGUUGUGACGUACA[i]	−11.8	50.6	−14.0	52.7	2.2	−2.1
UAUACAAGUUAUCUA[i]	−7.8	35.9	−7.9	36.5	0.1	−0.6
UAGAUAACUUGUAUA[i]	−7.7	35.2	−8.4	38.0	0.7	−2.8
CGACUAUGCAAAAAC[i]	−11.3	47.3	−12.8	50.1	1.5	−2.8
GUUUUUGCAUAGUCG[i]	−8.7	39.0	−9.8	41.8	1.1	−2.8
CGCAAAAAAAAAACGC[i]	−13.0	50.2	−13.8	51.4	0.8	−1.2
GCGUUUUUUUUUUGCG[i]	−5.9	28.7	−6.0	31.9	0.1	−3.2

[a]Hybrid sequences collected from previous studies (2–4). [b]All experiments were done in phosphate buffer (pH 7.0) containing 100 mM NaCl. [c]Melting temperatures were determined at the total oligomer strand concentration of 8 μM. [d]Stabilities of all hybrid duplexes were predicted using new nearest-neighbour parameters (Table 2). [e]$\Delta\Delta G°_{37}$ and [f]ΔT_m were calculated using same equation as defined in Table 1. The average value of $\Delta\Delta G°_{37}$ and ΔT_m in 100 mM NaCl for 26 sequences are obtained 8.5 % and 2.7 °C, respectively. [g]Sequences collected from Nakano *et al.* report (2). [h]Sequences taken from the report by Gyi *et al.* (3). [i]Sequences selected from the report by Lesnik and Freier (4).

Table S5. Thermodynamic parameters measured and predicted for RNA/DNA hybrids in 100 mM KCl buffer solution

No.	RNA Sequences[a]	Measured parameters in 100 mM KCl buffer[b]				Current prediction[d]	
		$\Delta H°$ /kcal mol^{-1}	$T\Delta S°$ /kcal mol^{-1}	$\Delta G°_{37}$ /kcal mol^{-1}	T_m[c] /°C	$\Delta G°_{37}$ /kcal mol^{-1}	T_m[c] /°C
9	GUUCAAUACG	−64.5 ± 1.8	−59.0 ± 1.7	−5.5 ± 0.3	26.0	−6.3	29.6
10	AGGAUGACCG	−78.6 ± 3.2	−68.5 ± 2.8	−10.1 ± 0.5	45.0	−10.9	48.0
17a	GGCAGGAAUCCG	−113.5 ± 1.2	−99.0 ± 1.2	−14.5 ± 0.2	55.7	−14.2	56.5
18a	UAUCUUCCGAAU	−76.8 ± 2.5	−70.3 ± 2.3	−6.5 ± 0.3	30.4	−7.0	32.9

[a]Four RNA sequences were selected from Table 1. [b]Thermodynamic parameters were measured in a solution containing 100 mM KCl, 10 mM K_2HPO_4, and 1 mM K_2EDTA (pH 7.0). [c]Melting temperatures were determined at the total oligomer strand concentration of 8 μM. [d]Stabilities were predicted using the new parameters (Table 2). The average value of $\Delta\Delta G°_{37}$ and ΔT_m were calculated at 7.3% and 2.4 °C, respectively.

Table S6. Thermodynamic parameters measured for RNA/DNA hybrids in 100 mM NaCl buffer solution with and without 1 mM $MgCl_2$

No.	RNA Sequences[a]	Measured parameters with 1 mM $MgCl_2$ in 100 mM NaCl [b]				Measured stabilities without $MgCl_2$[d]	
		$\Delta H°$ /kcal mol^{-1}	$T\Delta S°$ /kcal mol^{-1}	$\Delta G°_{37}$ /kcal mol^{-1}	T_m[c] /°C	$\Delta G°_{37}$ /kcal mol^{-1}	T_m[c] /°C
17a	GGCAGGAAUCCG (Group A)	−113.4 ± 4.5	−97.7 ± 4.1	−15.7 ± 0.8	59.2	−14.2 ± 0.5	56.8
21	GGCUCAAUUGAC (Group C)	−102.5 ± 1.4	−91.2 ± 1.3	−11.3 ± 0.2	46.8	−10.7 ± 0.3	45.2
23	UCCGAAUUAUCU (Group B)	−83.4 ± 2.2	−75.1 ± 2.0	−8.3 ± 0.3	38.0	−7.9 ± 0.4	35.8

[a]Three RNA sequences were selected from Table 1 that belong to three different groups of hybrid sequences (Group A, Group C, and Group B, respectively). [b] Thermodynamic parameters were measured in a solution containing 1 mM $MgCl_2$, 100 mM NaCl, and 10 mM Na_2HPO_4 (pH 7.0). [c]Melting temperatures were determined at the total oligomer strand concentration of 8 μM. [d] Stabilities were measured in a buffer containing 100 mM NaCl (Table 1).

Table S7. Comparison of sgRNA binding affinity based on stability predicted using old and new parameters with cleavage efficiency

sgRNA target site[a]	sgRNA[b]	$\Delta G°_{37}$ /kcal mol^{-1} (in 100 mM NaCl)		Efficiency[e]
		by new parameters[c]	by old parameters[d]	(%)
17-sgGFP86	GTGTCCGGCGAGGGCGA	−24.9	−19.2	>95[f]
17-sgGFP150	CACGGGCAGCTTGCCGG	−22.4	−19.1	>95[f]
17-sgGFP101	GAGGGCGATGCCACCTA	−21.3	−17.7	>95[f]
17-sgGFP198	GAAGCACTGCACGCCGT	−20.8	−16.9	>95[f]
17-sgGFP42	GTCCAGCTCGACCAGGA	−21.2	−16.5	>95[f]
17-sgGFP53	CTGGACGGCGACGTAAA	−20.1	−16.5	>95[f]
17-sgGFP261	GTAGCCTTCGGGCATGG	−20.0	−17.0	>95[f]
17-sgGFP544	GGGTGTTCTGCTGGTAG	−19.7	−15.7	>95[f]
17-sgGFP591	GCTCAGGTAGTGGTTGT	−19.3	−14.7	>90[f]
17-sgGFP379	GGGCATCGACTTCAAGG	−19.1	−16.2	>90[f]
17-sgGFP228	GTGCTGCTTCATGTGGT	−18.5	−14.4	~90[f]
17-sgGFP226	GCTGCTTCATGTGGTCG	−18.3	−14.7	~90[f]
17-EGFP site 3	GGTGCAGATGAACTTCA	−17.8	−14.0	~80[g]
17-sgGFP132	GGTGCAGATGAACTTCA	−17.8	−14.0	~80[f]
17-EGFP site 2	GCCGTTCTTCTGCTTGT	−14.5	−12.9	~60[g]
17-*in vitro*-matched	GCGAACTATACAACCTA	−14.4	−13.3	~35[h]
Comparisons for sgRNA of lengths other than 17 mer				
20-EGFPgRNA	GGCGAGGGCGATGCCACCTA	−27.0	−22.3	>95[g]
19nt-EGFPgRNA	GCGAGGGCGATGCCACCTA	−24.7	−20.4	>95[g]
18-EGFP site 1	GCACGGGCAGCTTGCCGG	−24.4	−20.8	>95[g]
20-EGFP site 3	GGTGGTGCAGATGAACTTCA	−23.1	−17.5	>95[g]
18-EGFP site 4	GCGAGGAGCTGTTCACCG	−21.7	−17.5	>80[g]
20-EGFP site 2	GATGCCGTTCTTCTGCTTGT	−18.0	−15.3	~55[g]
20-*in vitro*-matched	GCGAACTATACAACCTACTA	−16.8	−15.5	~55[h]
18-*in vitro*-matched	GCGAACTATACAACCTAC	−15.9	−14.6	~50[h]
16-EGFP site 3	GTGCAGATGAACTTCA	−15.5	−12.2	<5[g]
16-sgGFP132	GTGCAGATGAACTTCA	−15.5	−12.2	<5[f]
13-EGFPgRNA	GCGATGCCACCTA	−13.9	−12.1	<5[i]
15-*in vitro*-matched	GCGAACTATACAACC	−13.5	−12.3	<5[h]

[a]Target sites for the knockout experiments in CRISPR-Cas9 technique. [b]Guide RNA (sgRNA) with short to full length selected from previous studies. [c]Stability in physiological condition predicted with the derived new NN parameters (Table 2). [d] Stability in physiological condition predicted by linear equation (2) and the parameters obtained in 1 M NaCl solution (1). [e]Knockout efficiency for corresponding sgRNA collected from the reports given here as [f], [g], [h], and [i]. [f]*Sci. Rep.* 2016, 6, 1-10 (5). [g]*Nat. Biotechnol.* 2014, 32, 279-284 (6). [h]*Chem. Sci.* 2016, 7, 4951-4957 (7). [i]*J. Vet. Sci.* 2019, 20, e23 (8).

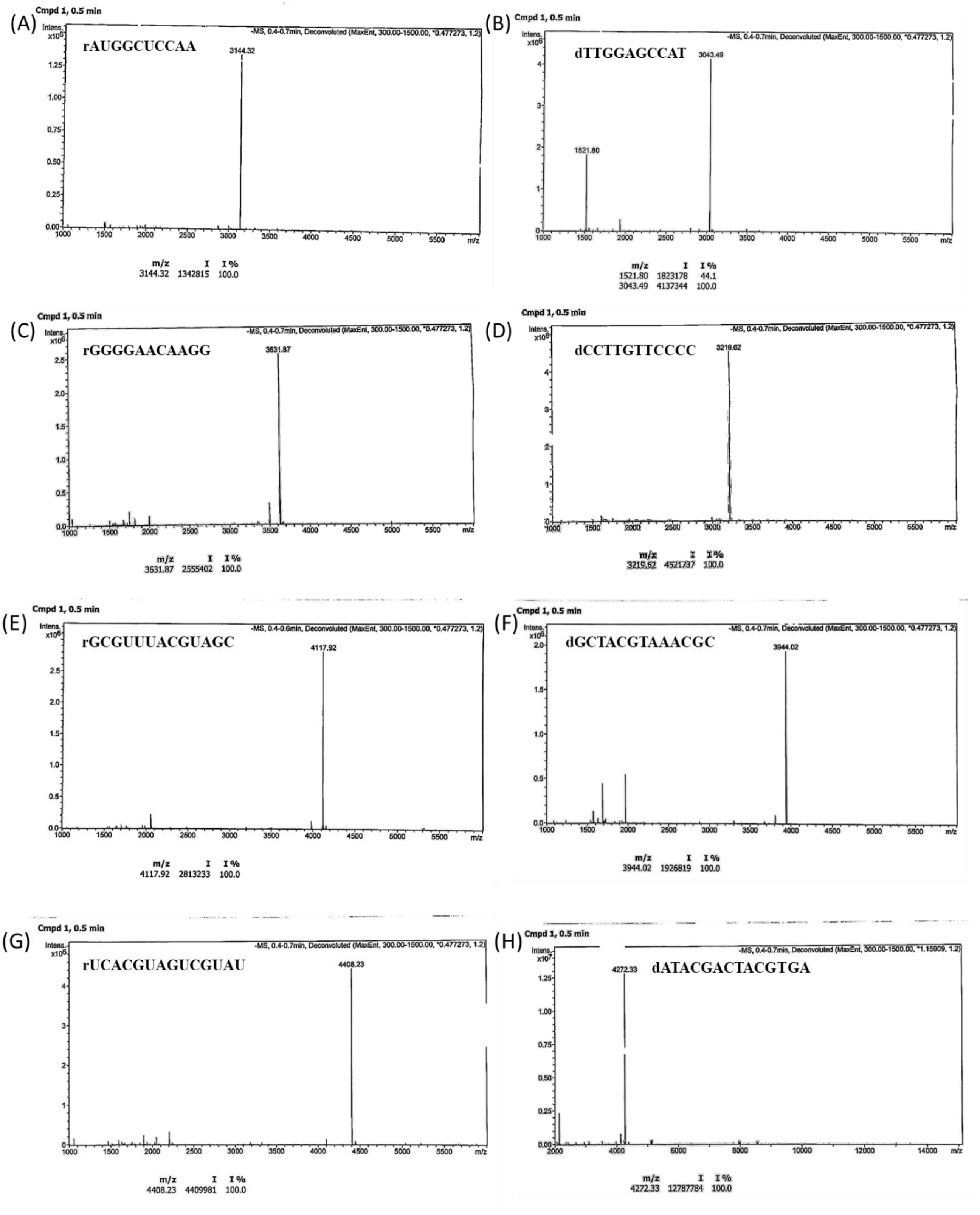

(**Figure S1** continued)

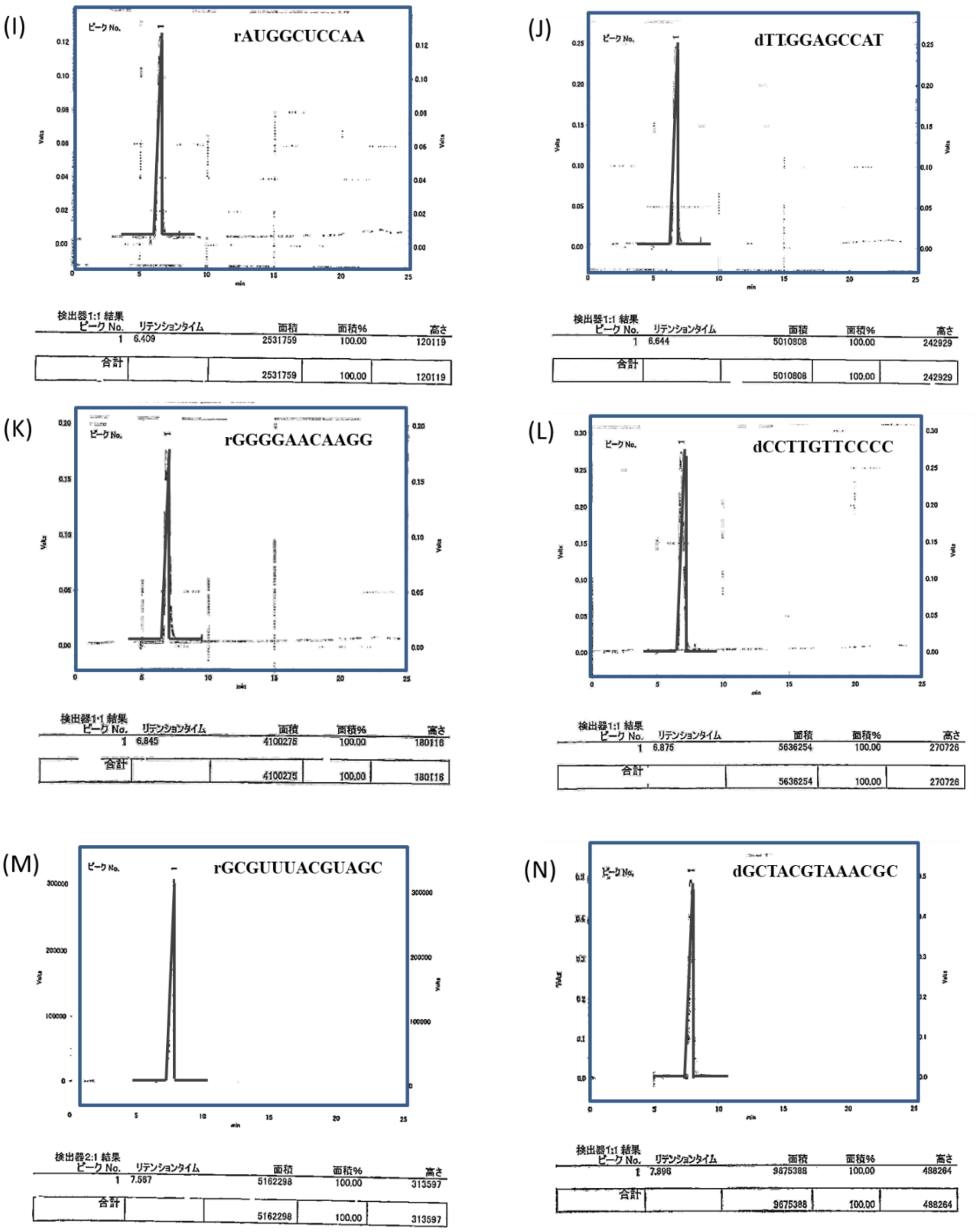

(**Figure S1** continued)

(O)

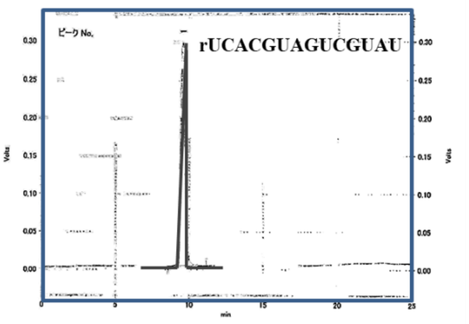

検出器1:1 結果

ピーク No.	リテンションタイム	面積	面積%	高さ
1	9.555	6222721	100.00	295092
合計		6222721	100.00	295092

(P)

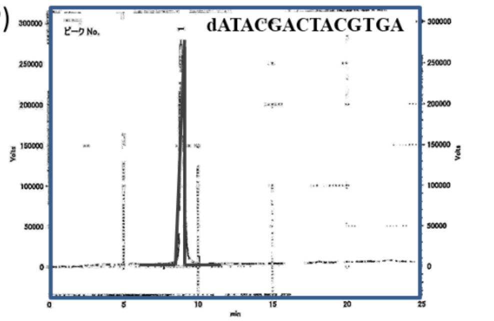

検出器2:1 結果

ピーク No.	リテンションタイム	面積	面積%	高さ
1	8.905	5612083	100.00	277463
合計		5612083	100.00	277463

Figure S1. MS (A-H) and HPLC (I-P) of the four RNA and complementary DNA oligonucleotides of four model hybrid sequences written from 5´ to 3´.

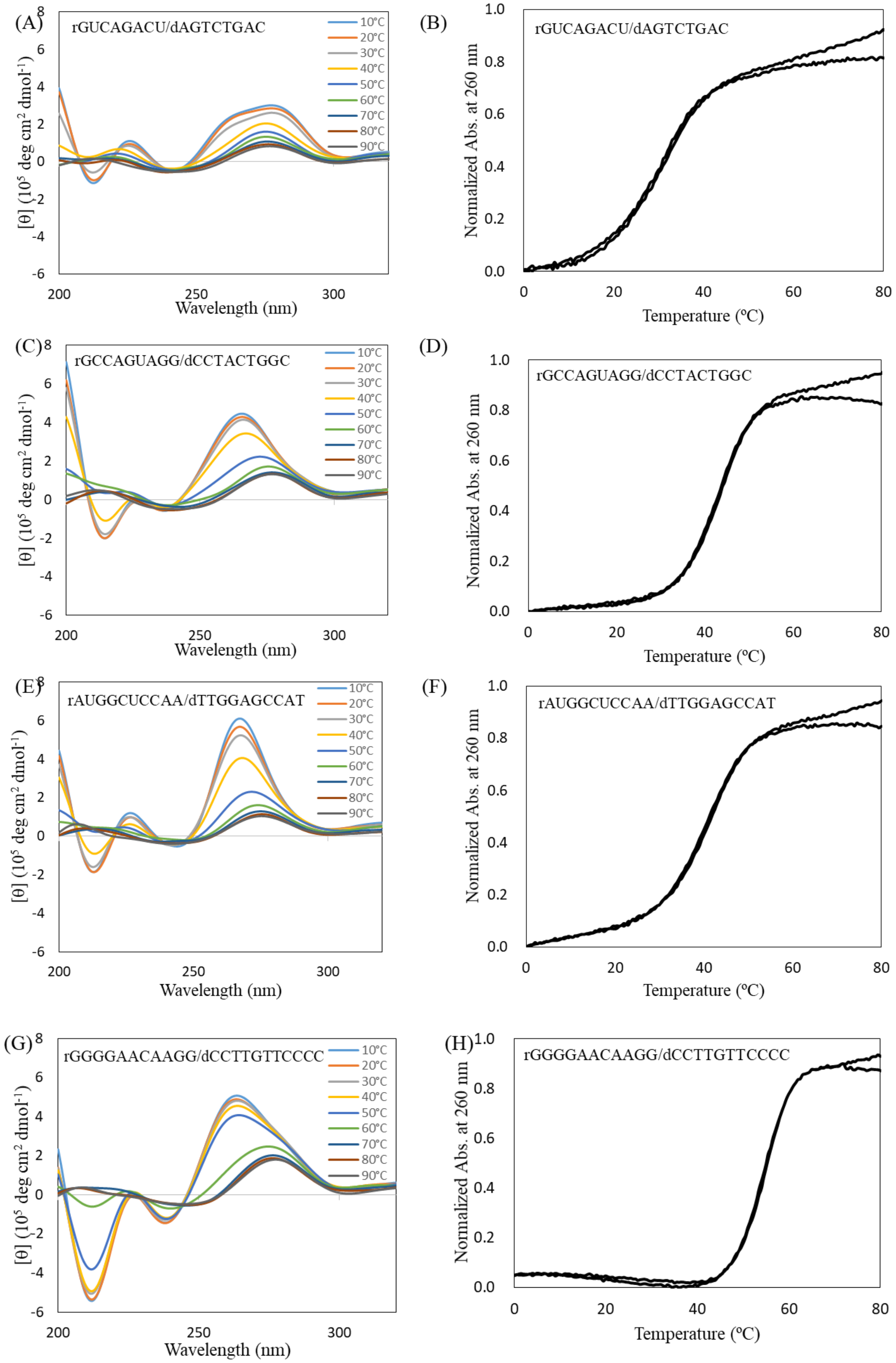

(Figure S2 continued**)**

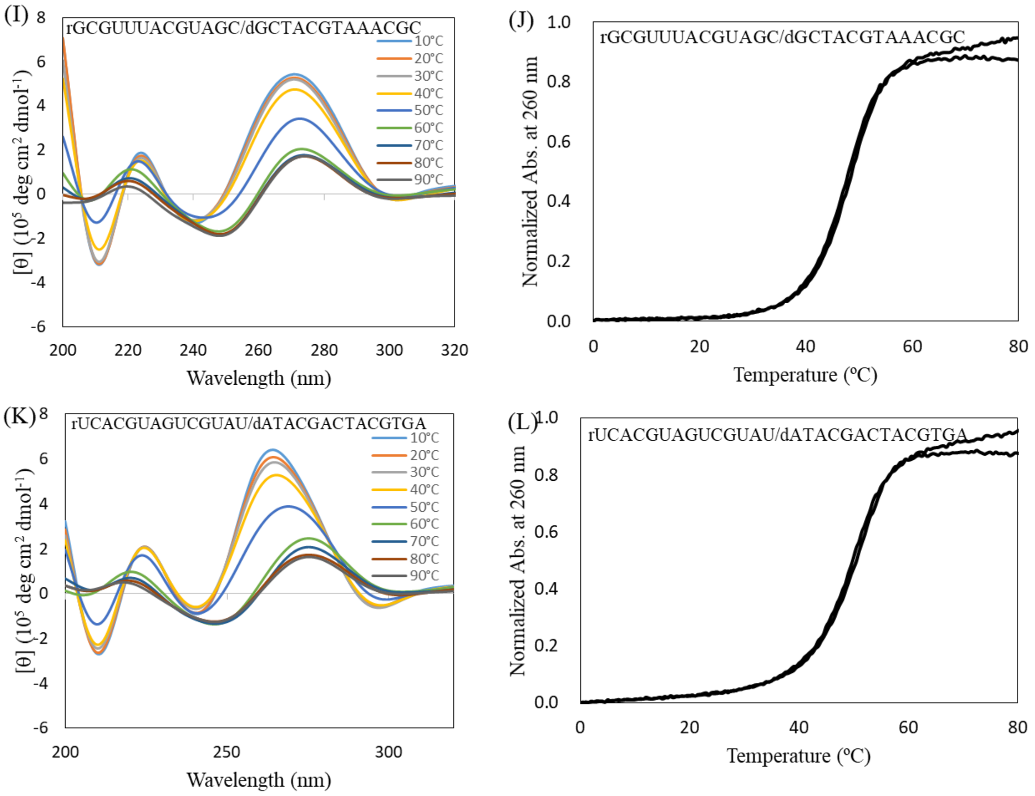

Figure S2. Temperature induced unfolding CD assays (A, C, E, G, I, and K) and UV-melting curves of denaturation and renaturation (B, D, F, H, J, and L, respectively) for 6 model hybrid sequences having different oligomer lengths and base compositions.

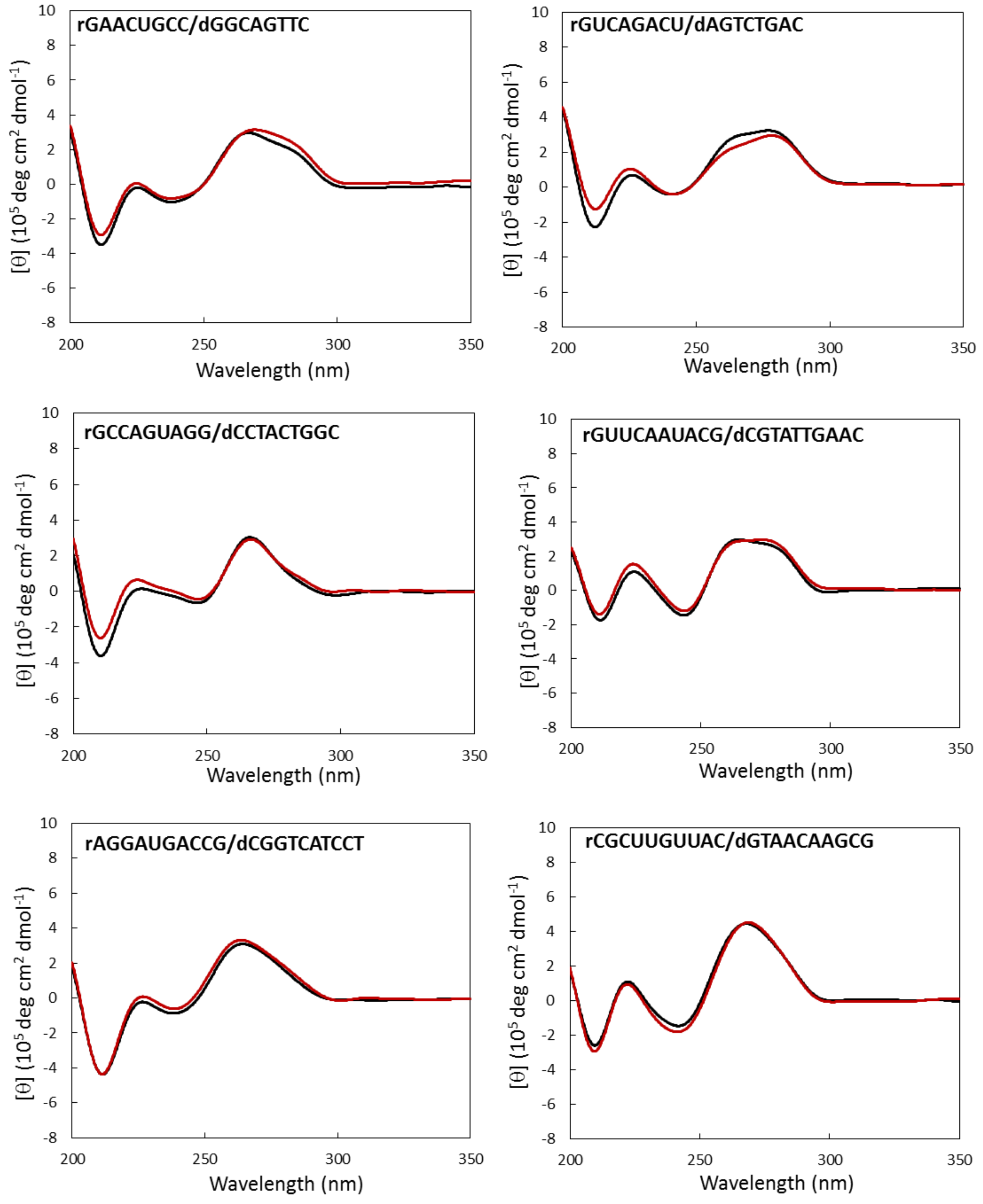

(Figure S3 continued**)**

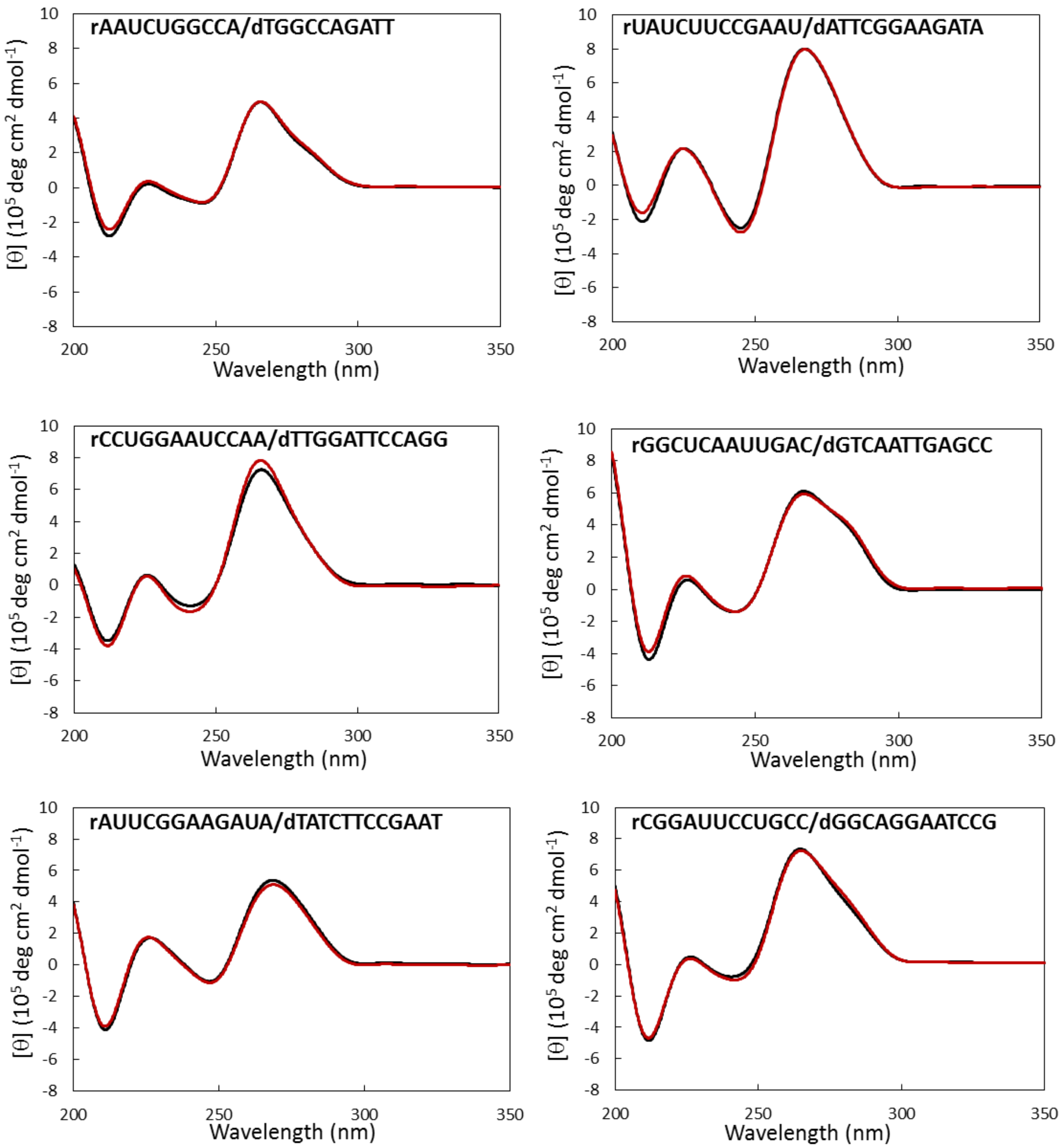

Figure S3. CD spectra of respective RNA/DNA hybrids selected from Table 1 in buffer solutions containing 1 M (black) and 100 mM (red) NaCl using 20 μM of total strand concentration at 4 °C.

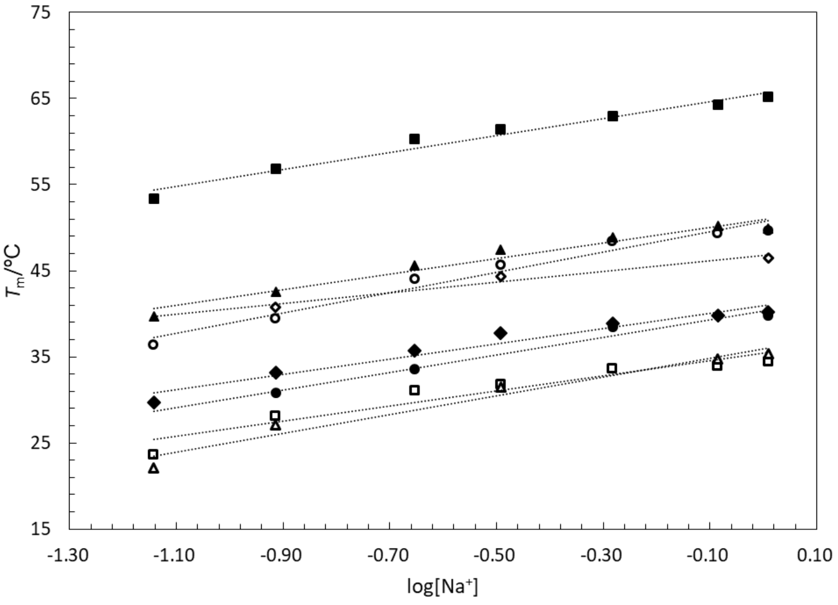

Figure S4. Melting temperatures were measured at the total strand concentration of 8 μM in buffer solution (pH 7.0) containing 10 mM Na_2HPO_4, 1 mM Na_2EDTA and different concentration (from 1 M to 50 mM) of NaCl and plotted vs log[Na^+] for the representative 8 RNA/DNA hybrid duplexes of different base compositions. The RNA/DNA hybrids and their sequence contents are written here with the slope values of corresponding linear plots in parentheses - (◊) 75% rG–dC or rC–dG rich rGCCGUGAG/dCTCACGGC with 63% purine rich RNA (6.2); (▴) 67% rG–dC or rC–dG rich rGCCAGUAGG/dCCTACTGGC with 67% purine rich RNA (9.1); (□) 60% rA–dT or rU–dA rich rCACUUGUUAC/ dGTAACAAGTG with 70% pyrimidine rich RNA (8.7); (Δ) 60% rA–dT or rU–dA rich rGUUCAAUACG/dCGTATTGAAC having 50% purine in RNA (10.9); (♦) 50% rG–dC or rC–dG containing rCGCUUGUUAC/dGTAACAAGCG with 70% pyrimidine rich RNA (8.9); (o) 50% rG–dC or rC–dG containing rAUGGCUCCAA/dTTGGAGCCAT with 50% purine in RNA (11.8); (▪) 67% rG–dC or rC–dG rich rGGCAGGAAUCCG/dCGGATTCCTGCC with 67% purine rich RNA (9.8); (•) 67% rA–dT or rU–dA rich rUAUCUUCCGAAU /dATTCGGAAGATA with 67% pyrimidine rich RNA (10.2).

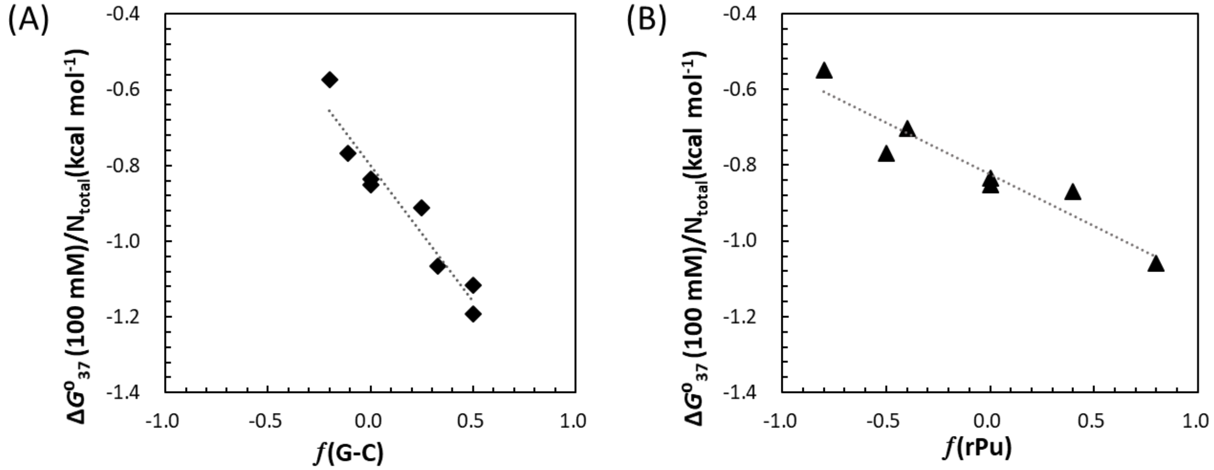

Figure S5. The plot of measured ΔG°_{37} per base pair in 100 mM NaCl against f(G–C) for the sequence of set 2 with f(rPu) = 0 (A), and against f(rPu) for the sequence of set 1 with f(G–C) = 0 (B).

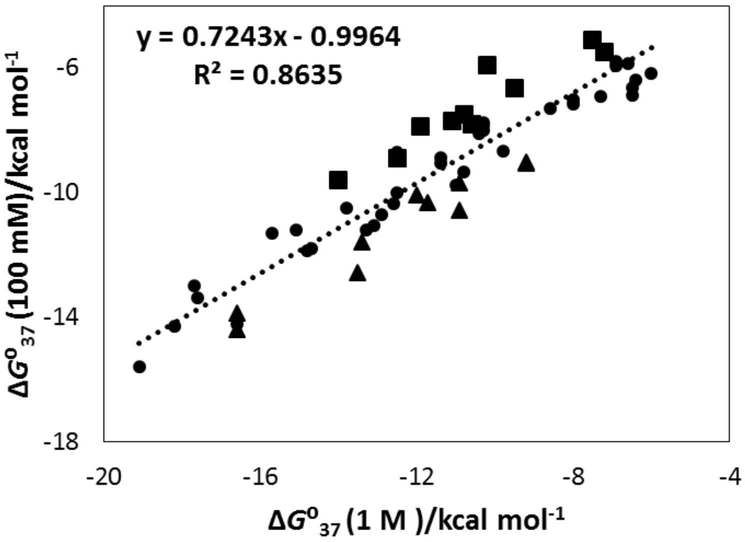

Figure S6. The plot of measured $\Delta G°_{37}$ in 100 mM NaCl vs the predicted $\Delta G°_{37}$ in 1 M NaCl using the data of our sequences and previously reported sequences[1, 3] altogether where the hybrid duplexes of Group A were denoted as the symbol (▲), hybrids of Group B (■), and hybrid sequences of Group C (●) same as Figure 2.

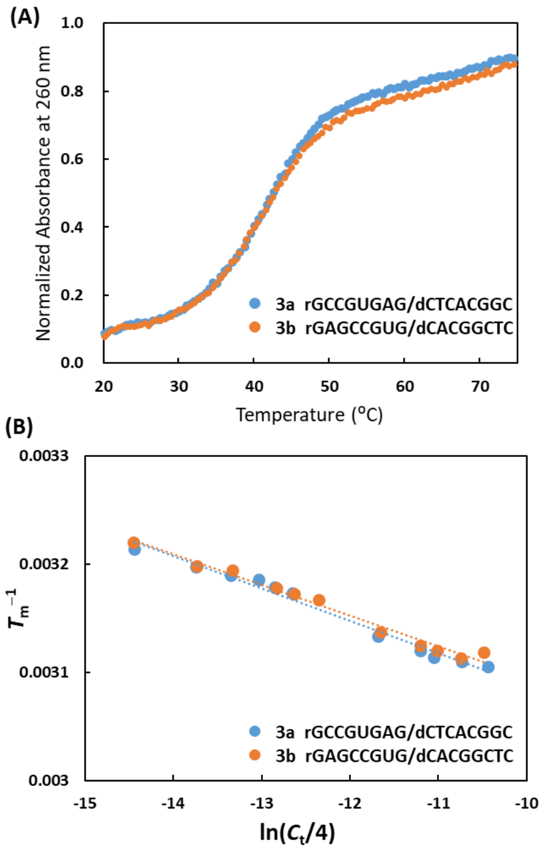

Figure S7. (A) Normalized UV melting curves of duplex pairs 3a rGCCGUGAG/dCTCACGGC (blue) and 3b rGAGCCGUG/dCACGGCTC (orange) having identical nearest neighbors in the buffer containing 100 mM NaCl. The total concentration of oligonucleotides was 8μM; (B) T_m^{-1} versus $\ln(C_t/4)$ plots of 3a and 3b.

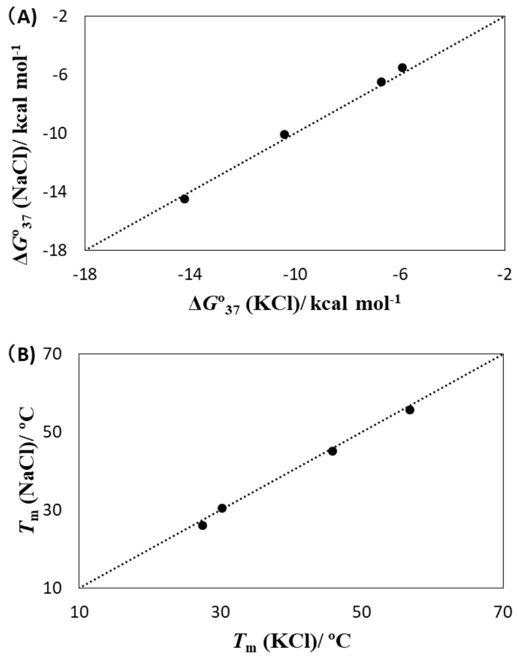

Figure S8. The plot of measured ΔG°_{37} (A) and T_m (B) in 100 mM NaCl vs that in 100 mM KCl using the data of four sequences of Table S5.

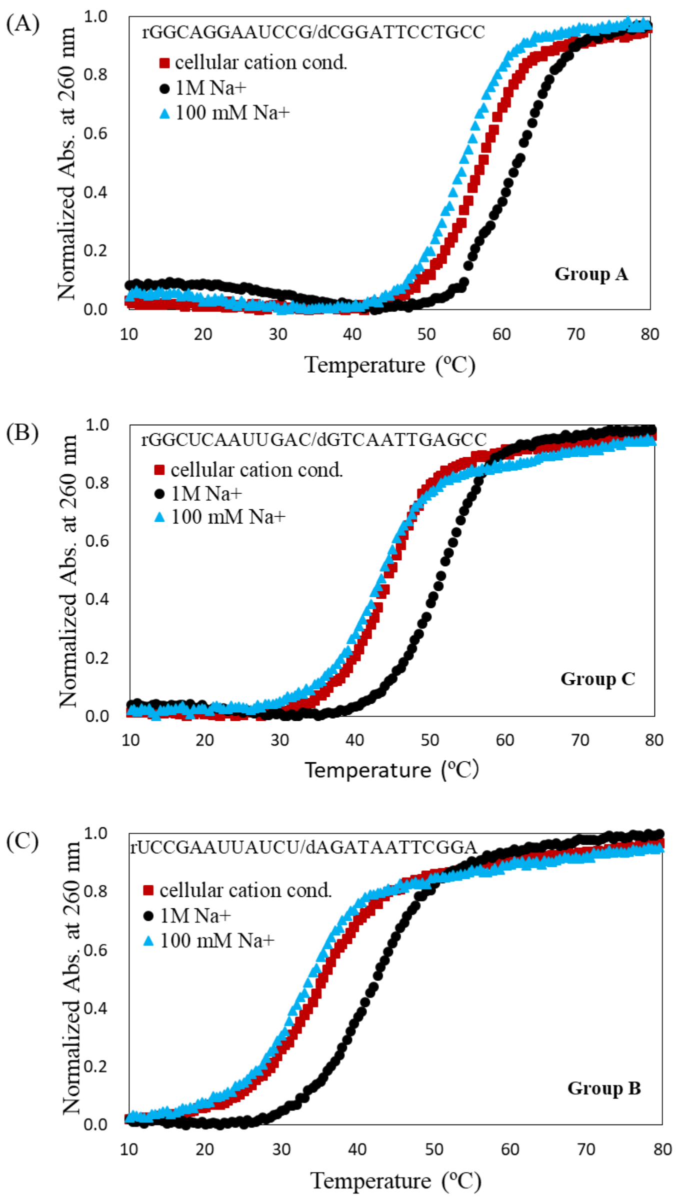

Figure S9. Normalized UV melting curves in a physiological salt solution (■), 1 M (●), and 100 mM (▲) NaCl solution of hybrid duplexes from Group A (A), Group C (B), and Group B (C), respectively.

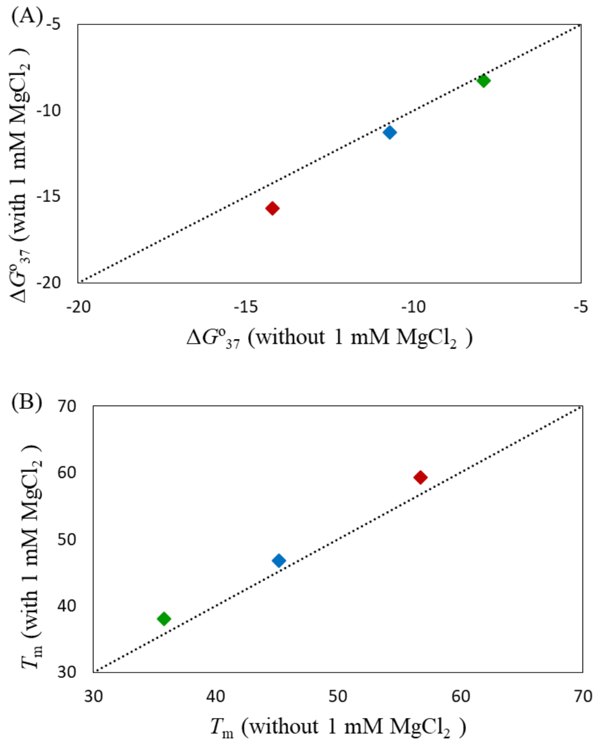

Figure S10. The plot of measured $\Delta G°_{37}$ (A) and T_m (B) in presence versus in absence of 1 mM $MgCl_2$ in a buffer containing 100 mM NaCl for the three sequences of Table S6 belonging to three different groups (Group A (red), Group B (green), and Group C (blue)).

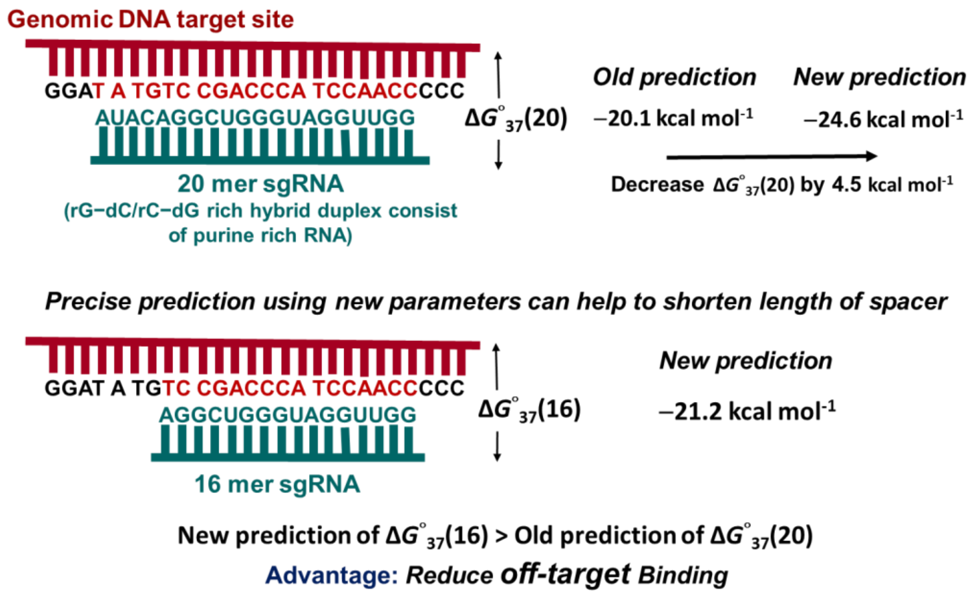

Figure S11. Schematic presentation about the advantage of the precise prediction for hybrid duplex in CRISPR-Cas9 gene-editing technique.

References:

1. Sugimoto,N., Nakano,S., Katoh,M., Matsumura,A., Nakamuta,H., Ohmichi,T., Yoneyama,M. and Sasaki,M. (1995) Thermodynamic Parameters To Predict Stability of RNA/DNA Hybrid Duplexes. *Biochemistry*, **34**, 11211–11216.

2. Nakano,S., Fujimoto,M., Hara,H. and Sugimoto,N. (1999) Nucleic acid duplex stability: Influence of base composition on cation effects. *Nucleic Acids Res.*, **27**, 2957–2965.

3. Gyi,J.I., Conn,G.L., Lane,A.N. and Brown,T. (1996) Comparison of the thermodynamic stabilities and solution conformations of DNA·RNA hybrids containing purine-rich and pyrimidine-rich strands with DNA and RNA duplexes. *Biochemistry*, **35**, 12538–12548.

4. Lesnik,E.A. and Freier,S.M. (1995) Relative Thermodynamic Stability of DNA, RNA, and DNA:RNA Hybrid Duplexes: Relationship with Base Composition and Structure. *Biochemistry*, **34**, 10807–10815.

5. Zhang,J.P., Li,X.L., Neises,A., Chen,W., Hu,L.P., Ji,G.Z., Yu,J.Y., Xu,J., Yuan,W.P., Cheng,T., *et al.* (2016) Different Effects of sgRNA Length on CRISPR-mediated Gene Knockout Efficiency. *Sci. Rep.*, **6**, 1–10.

6. Fu,Y., Sander,J.D., Reyon,D., Cascio,V.M. and Joung,J.K. (2014) Improving CRISPR-Cas nuclease specificity

using truncated guide RNAs. *Nat. Biotechnol.*, **32**, 279–284.

7. Zhang,K., Deng,R., Li,Y., Zhang,L. and Li,J. (2016) Cas9 cleavage assay for pre-screening of sgRNAs using nicking triggered isothermal amplification. *Chem. Sci.*, **7**, 4951–4957.

8. Lv,J., Wu,S., Wei,R., Li,Y., Jin,J., Mu,Y., Zhang,Y., Kong,Q., Weng,X. and Liu,Z. (2019) The length of guide RNA and target DNA heteroduplex effects on CRISPR/Cas9 mediated genome editing efficiency in porcine cells. *J. Vet. Sci.*, **20**, 3–11.
